# Differential C3NET reveals disease networks of direct physical interactions

**DOI:** 10.1186/1471-2105-12-296

**Published:** 2011-07-21

**Authors:** Gökmen Altay, Mohammad Asim, Florian Markowetz, David E Neal

**Affiliations:** 1 Department of Oncology, University of Cambridge, Cambridge Research Institute, CB2 0RE, Cambridge, UK; 2 Cancer Research UK Cambridge Research Institute, Li Ka Shing Centre, CB2 0RE, Cambridge, UK

## Abstract

**Background:**

Genes might have different gene interactions in different cell conditions, which might be mapped into different networks. Differential analysis of gene networks allows spotting condition-specific interactions that, for instance, form *disease networks *if the conditions are a disease, such as cancer, and normal. This could potentially allow developing better and subtly targeted drugs to cure cancer. Differential network analysis with direct physical gene interactions needs to be explored in this endeavour.

**Results:**

C3NET is a recently introduced information theory based gene network inference algorithm that infers direct physical gene interactions from expression data, which was shown to give consistently higher inference performances over various networks than its competitors. In this paper, we present, DC3net, an approach to employ C3NET in inferring disease networks. We apply DC3net on a synthetic and real prostate cancer datasets, which show promising results. With loose cutoffs, we predicted 18583 interactions from tumor and normal samples in total. Although there are no reference interactions databases for the specific conditions of our samples in the literature, we found verifications for 54 of our predicted direct physical interactions from only four of the biological interaction databases. As an example, we predicted that RAD50 with TRF2 have prostate cancer specific interaction that turned out to be having validation from the literature. It is known that RAD50 complex associates with TRF2 in the S phase of cell cycle, which suggests that this predicted interaction may promote telomere maintenance in tumor cells in order to allow tumor cells to divide indefinitely. Our enrichment analysis suggests that the identified tumor specific gene interactions may be potentially important in driving the growth in prostate cancer. Additionally, we found that the highest connected subnetwork of our predicted tumor specific network is enriched for all proliferation genes, which further suggests that the genes in this network may serve in the process of oncogenesis.

**Conclusions:**

Our approach reveals disease specific interactions. It may help to make experimental follow-up studies more cost and time efficient by prioritizing disease relevant parts of the global gene network.

## Background

**Figure 1:**
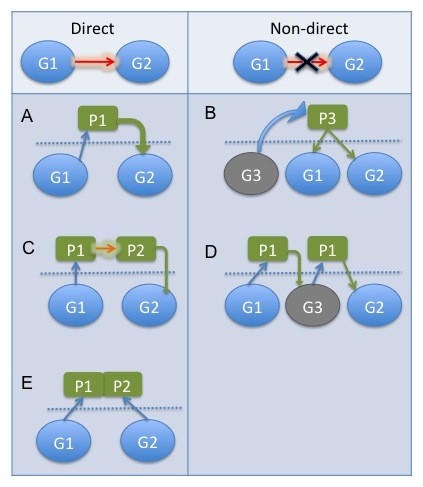
**Examples of direct physical gene interactions**. Examples of direct (A, C, E) and non-direct (B, D) physical gene interactions. G, P are for gene and protein (or transcription factor), respectively. Direct: Fig 1A. G1 encodes P1 that directly regulates G2, Fig 1C. G1 encodes a kinase protein that phosphorylates P2 that regulates G2, Fig 1E. G1 encodes P1 that makes protein complex with P2 that is also encoded by G2. Non-direct: Fig 1B. G3 encodes P3 that regulates both G1 and G2, Fig 1D. G1 encodes P1 that regulates G3 that encodes P3 that regulates G2.

Treatment of a disease, such as cancer, requires understanding of normal cell physiology and pathogenesis [1-3]. This goal is extremely difficult as cellular biomolecules are constantly interacting with each other in a dynamic manner. These interactions are often represented as networks. Depending on the biological level of interactions, the networks can be classified [4] as metabolic networks, gene networks, protein networks, and so on. The biological networks may also be classified according to the type of interaction under consideration such as direct physical gene interaction networks and associative gene networks. By *direct physical gene interaction *we mean the interaction between a gene pair that is not mediated by a third gene of the dataset under consideration. In Figure 1, we have illustrated some of the examples of direct and non-direct physical gene interactions with explanations in the caption. Basically, two genes are considered to be directly physically interacting if they express together with no involvement of a third gene between them, considering the fact that any other molecules might be involved in the molecular process. In fact, this definition is a result of the limitations inherent in gene expression datasets, in which only mRNA expressions are available. Since we do not have information about any other molecule (protein, metabolites, etc.) other than genes, we have to map a network of genes by ignoring other molecules. The difference of direct physical interaction regarding associative networks is the specification of direct interaction of genes among them rather than a group of coexpressed genes with any possible order of molecular reactions among the genes. By associative network we refer to the weighted gene coexpression networks [5], where there is measurable relation between the gene pair but the relation may well be over a third or other genes. The task of inferring direct physical interactions is more difficult to fulfil. Because in a very complex and large network of interactions, for a gene pair, it is more likely to predict an indirect interaction from gene expression data as the indirect interactions also get very close correlation values. Moreover, the number of direct interactions are dramatically low considering the whole set of possible interactions in real expression datasets. Despite the difficulty of this task, it is of highest importance for drug development as it specifies direct targets of gene interactions of interest.

Inferring gene networks of direct physical interactions *in vivo *or *in vitro *via laboratory experiments provide accurate detections, but it is very labour intensive process and limited by the number of interactions that can be detected experimentally [1]. However, current biotechnology produces large-scale microarray gene expression datasets that can be used in computational methods to supplement biochemical screens for interaction partners. Nowadays, it is typical to come across, for example, a *homo sapiens *expression dataset with around 25000 genes and 1000 samples. Working on the probe-level for more resolution increases this number significantly. The size might even be much higher when working on the intron and exon levels such as around 600 thousands of probes. Interpreting this abundance of datasets requires powerful computational reverse engineering algorithms and tools. Gene network inference (GNI) algorithms showed significance advance in this endeavour [6].

Among GNI algorithms, *information theory *based ones [1,7-10] are computationally feasible to implement on the very large-scale datasets with reasonable performances [6]. They use *mutual information *(MI) [11] as the measure of association between gene pairs. MI is superior to linear correlation measures, e.g. Pearson, as it is able to capture not only linear relationships between gene pairs but also nonlinear relationships. Among the information theory based methods, C3NET has been shown to give consistently best inference performances with low computational complexity [1] comparing other well known information theory based methods such as Relevance Network [7], ARACNE [8], CLR [9] and MRNET [10], and thus we employ it for the differential gene network (DGN) analysis approach of this study.

GNI algorithms predict gene networks with thousands of interactions from gene expression datasets but it is often difficult to interpret the resulting network itself. The large size often makes it look like a hairball. It is difficult for a biologist to spot the interaction or groups of interactions that are specifically related to the condition of interest, such as cancer. It is also reported that molecular interactions are dynamic with respect to different cell conditions and vast majority of interactions detected under one condition could not be detected under the other condition [12,13]. As an experimental example, 70% of positive genetic interactions, which are resulting in increased cell viability, under methyl methanesulfonate treatment were not identified in the untreated samples [12,13]. This biological phenomenon urges studies on the differential network level to exploit this difference. Differential network analysis is shown to be useful in filtering the networks to smaller size by comparing the pathways in different conditions such as non-recurrent primary prostate cancer and metastatic prostate cancer [14]. Specifically, the research field *disease networks *[15] is relatively new and a focal topic of interest. Here, by *disease network *we mean a network of biological interactions that only appears in the disease state of a cell but not in the normal state of a cell. This concept can be called more generically as *differential networks*, meaning the network of interactions that only appears in one condition but not in other condition or conditions. Considering the comparisons among multiple conditions leads to *differential network analysis*. A recent review on differential networking can be found in [4].

The methods developed for differential network analysis are mostly based on coexpression networks [16-28]. A short summary of them can be found in [27]. For example, a recent approach, called DiffCoEx [27] aims identifying gene coexpression differences between multiple conditions, which was developed based on Weighted Gene Coexpression Network Analysis (WGCNA) [5,29]. DiffCoEx detects differentially coexpressed modules using WGCNA. Nevertheless, it provides differentially coexpressed modules that are not necessarily to be direct physical interactions. Namely, DiffCoEx provides much more general results by showing significantly related genes in an associative way by clustering into gene modules.

In this paper, we focus on the inference for differential networks of only direct physical interactions by developing a comparison approach over C3NET. The developed approach infers only *direct physical interactions *of differential gene networks (DGN) from gene expression datasets of multiple conditions. The approach differs from others of [16-27] as it only provides direct physical interactions in the inferred network but not providing any kind of associative network or network modules. In order to make the paper easier to read, we call the presented approach as DC3net (Differential C3NET) for the rest of the paper. In general, DC3net infers the DGNs in multiple conditions and also provides common network of multiple conditions. Basically, having two cell conditions under consideration, such as test and control (e.g., tumor and normal), two gene networks are inferred by C3NET from the expression datasets and then compared with the decision filter between the two. Details of the decision filter can be seen in *DC3net elaborated *section.

Using the predicted DGN on the real prostate cancer dataset we further searched for tumor signatures with enrichment analysis. We spotted the highest connected subnetwork and discussed about its possible roles in prostate cancer. We also pointed out the oncogenes in the inferred differential and common networks that show the possible role of oncogenes in the predicted networks.

## Results and Discussion

### DC3net overview

**Figure 2:**
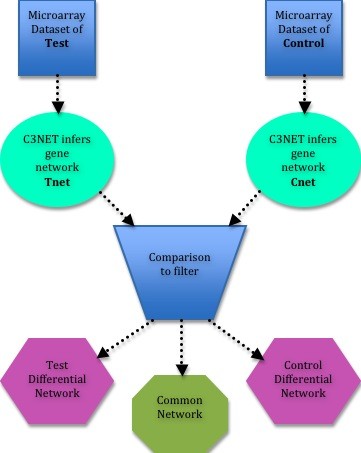
**DC3net overview**. An outline of the approach DC3net with main processes.

The motivation to introduce DC3net is to find the direct physical gene interactions that appear only in disease related cells but do not appear in normal healthy cells so that we can infer only disease network of genes and eliminate most of the interactions that is not related to the disease of interest. The same approach can be applied to any two different conditions cells to spot the different and common gene interactions between them. In the figure, the statistical term *test *might refer to tumor samples and *control *to normal samples as we have used in our exemplary prostate cancer dataset. As can be seen in Figure 2, we provide three different networks as output, where the most important one is the test *differential network *(*difnet*) that incorporates only the interactions of disease condition, which we call as *disease network*. Second one is the *common network *of the interactions of both conditions. This network shows the essential interactions required for both conditions and is a much more accurately predicted network, as the interactions are the overlapping links that are inferred independently in both cases. Third one is the *control difnet*, which consists of interactions that appears in the control case but not in the test case. It is also quite important as it shows the required interactions of healthy cell, which disappears in tumor case.

In order to be consistent and compact along the paper we call all the networks of Figure 2 as follows: The networks that are inferred from test and control microarray data are called as *Tnet *and *Cnet *respectively. We call the three output networks with more generic names as this approach can also be applied to two different tumor types of samples to see the differences among them. Therefore we call them as *test difnet*, *common network*, and *control difnet*, respectively as seen in Figure 2. We keep italicise them to avoid confusion.

The details of DC3net will be presented in section of *DC3net elaborated*. Here, we give a brief explanation to the outline of DC3net as illustrated in its simplest form in Figure 2 for more general audience: Two different microarray gene expression data sets, one as test (e.g. tumor case) and the other as control (e.g. normal or healthy) are used as input. Applying C3NET to each of the data sets, two different gene networks of direct physical interactions are inferred, which are called as *Tnet *and *Cnet*, respectively. By comparing these two networks in the decision filtering step, first the *common network *is inferred by selecting all the overlapping interactions between the networks. Then *test difnet *is determined by selecting the interactions of *Tnet *that do not have strong correlation values in the control case. Similar process is performed to determine *control difnet *with respect to the test correlation matrix. The details of all the steps can be found in section of *DC3net elaborated*.

It is worth mentioning that, although we compare two different networks of two different conditions, it is pretty straightforward to apply DC3net to three or more datasets of different conditions if required.

### Performance of DC3net and biological validations

In this section, we discuss the application results obtained using DC3net on a real prostate cancer dataset. Before applying DC3net on the real dataset, we performed analysis on a synthetic dataset to get an approximate idea on the inference performance of DC3net. We observed that the inference results on the synthetic datasets are very promising. In order not to distract the reader's attention from the results of the real application, we present the details of the implementation and results of the synthetic dataset in Additional File 1.

Applying DC3net on the prostate cancer and normal datasets, we inferred the tumor (test) network, *Tnet*, with 9653 interactions and the normal (control) network, *Cnet*, with 8930 interactions. We inferred *tumor difnet *with 2409 interactions, *normal difnet *with 2025 interactions and *common network *with 992 interactions, where all the interactions of these networks are provided in a tab delimited file format in the Additional File 2. The Excel file is very useful to search some specific genes of interest and also sorting them with respect to MI weight or gene name as required. Nevertheless, the visualisations of these large networks are not readable when illustrated in a single page. Therefore we plot only some of the interesting subnetworks from these networks, which are all combined in a single *pdf *file and available in Additional File 3. Visualisation of these networks brings additional information, because the highly connected subnetworks are not perceivable from the Excel file. For example we plot top 250 genes with respect to MI weight representing the strongest interactions. We also plot, for instance, the hubs with order greater than 3 and 2 to spot the hub genes. From that, as an example, we have observed that the gene NONO appears to be as a hub in normal *difnet *but disappears in tumor *difnet*. Similar observations can be spotted by a visual inspection among the plotted networks of Additional File 3.

In order to find biological verifications to support our predicted interactions from the literature, we searched some of the suitable databases such as Human Protein Reference Database (HPRD) [30], BioGrid [31], ID-Serve database in supplementary file 4 of [32] and also the B cell interactome (BCI) [33,34]. We found 54 unique interactions (5 in *tumor difnet*, 3 in *normal difnet *and 46 in *common network*) verified by the databases, which confirms some of the predictions of C3NET. The numbers of verifications regarding the biological databases in which they are found are tabulated in Table 1. We only considered the experimentally verified interactions of BCI as it also includes the predictions from which we would have had much higher number of supports from the whole set of it. The verified unique interactions and which network category they belong to are provided in Table 2. While searching for verifications, we have directly looked up with respect to the gene names that we have and also the gene names of the databases. The number of verifications might be higher as genes have in fact many different aliases. There are also many other databases in which some more verification might be found. Moreover, we did not see any specific database for prostate cancer gene interactions that we could use as a reference interactions database. However, our general literature search is performed thoroughly in those four databases.

**Table 1:** Number of validated predictions over various databases.

Databases	Tumor difnet	Normal difnet	Common network
HPRD	2	2	6
BioGrid	2	2	5
ID-Serve	2	1	42
BCI	11	7	37

Number of inferred direct physical interactions verified by public databases HPRD (Human Protein Reference Database), BioGrid, ID-Serve and BCI (B cell interactome).

**Table 2:** Validations from literature for the predictions.

Gene1	Gene2	Category
API5	DDX39	Tumor
MAPT	PPP5C	Tumor
TAP1	PSMB8	Tumor
TERF2	RAD50	Tumor
MYC	RPL3	Tumor

CCND1	NCOA3	Normal
TOB1	PABPC1	Normal
PRKG1	SF1	Normal

C1R	C1S	Common
MYC	CNTN2	Common
COL4A1	COL4A2	Common
UBC	CTNNB1	Common
EGR2	EGR3	Common
HLA-G	HLA-A	Common
HLA-G	HLA-F	Common
KLK3	KLK2	Common
DST	KRT5	Common
UBE2G2	MGRN1	Common
MT1E	MT1H	Common
MT1X	MT1H	Common
ESR1	POU4F1	Common
PSME2	PSMB1	Common
PSMB1	PSMB3	Common
PSMB4	PSMC1	Common
RPL15	RPL10A	Common
RPL22	RPL10A	Common
RPS17	RPL10A	Common
RPS13	RPL12	Common
RPS7	RPL12	Common
RPL11	RPL24	Common
RPS14	RPL29	Common
RPL12	RPL31	Common
RPL6	RPL31	Common
RPS18	RPL31	Common
RPS23	RPL31	Common
RPL27A	RPL34	Common
RPL24	RPL35	Common
RPL30	RPL35	Common
RPS12	RPL37	Common
RPS9	RPL8	Common
RPS12	RPL9	Common
RPS16	RPS11	Common
RPS8	RPS13	Common
RPL7	RPS15A	Common
RPL10	RPS2	Common
RPS20	RPS24	Common
RPL13	RPS28	Common
RPL23	RPS3A	Common
RPLP0	RPS4X	Common
RPL29	RPS5	Common
RPL27	RPS7	Common
RPL32	RPS7	Common
RPS6	RPS8	Common
COL1A2	SPARC	Common

The experimentally verified unique direct physical interactions from the databases and their predicted categories.

### The networks of oncogenes

We have also investigated the interactions of important genes in the prostate cancer data set. In this case study, the oncogenes are considered as important genes regarding the exemplary biological problem that is cancer. We downloaded the oncogene list [35] from [36], which consist of 436 oncogenes, and used it as a filter and then selected the interactions if any one of the genes of the gene pairs is in the list. We obtained subnetworks that can be considered as oncogene networks for three of the inferred differential networks. We plot these networks in Additional File 3. This helps determining the role of important genes in different conditions. From these analyses we observed that oncogenes are not only appearing with edges in tumor case but they also appear with similar number of interactions in normal cell with different gene partners. This implies that oncogenes might cause disease when they interact with different genes than normal case. We observed that 913 and 836 interactions with oncogenes available in *Tnet *and *Cnet *respectively. We also detected those 218, 192 and 110 interactions with oncogenes appear in *tumor difnet*, *normal difnet *and *common network*, respectively, which are provided in Additional File 3.

### Relation of DC3net with differential expression analysis

We also look at the relation between standard differential expression (DE) analysis [37] and our differential network analysis with DC3net. MI incorporates linear and non-linear relations and thus is a more general measure of correlation regarding linear correlation measures used for DE analysis. Therefore we expect, for example, top ranked DE gene with lowest p-value to appear in the tumor difnet but not necessarily with highest MI. Applying DE analysis in R [38] software package limma [37] to the prostate cancer dataset, we obtained gene HPN as the top ranked gene with p-value 4.41*10^-21 ^and adjusted p-value 5.57*10^-17^. Just as we expected, HPN appears in our inferred tumor *difnet *but with relatively lower MI value regarding other interactions. In tumor *difnet*, HPN has interactions with genes SYNGR2 and CYP27A1 having the ranks 1065 and 1696, out of 2410 interactions, with MI values 0.52 and 0.39 where the top ranked (rank 1) interaction has the MI value 1.33. We also look at the top ranked interaction of tumor *difnet *in DE genes. In general, the gene pairs of a differential interaction of tumor *difnet *is not necessarily to be DE genes as they might interact with different partners in different conditions and thus might not appear as DE gene. However, it is likely that at least one of them might also be DE gene. The top ranked interaction in tumor *difnet *is in between COL6A1 and PAX8. They have p-values of 0.00077 and 0.05 that supports our expectations, as they are reasonably low p-values but not among lowest. We also look at the lowest rank interaction of tumor *difnet*, which is in between LTBP2 and COL5A2. They have p-values of 0.006 and 0.004 that reflects our expectations as no strong relation exist from differential interactions to DE genes as we see top and lowest ranked interactions have similar p-values. This might be due to, in addition to algorithmic difference, the property of MI capturing also the non-linear effects upon the linear ones.

### Biological analysis of the predicted differential gene interactions

Significant amount of the interactions identified by DC3net have been shown to be already validated in the literature as shown in Table 2. These are the verifications considering only the direct physical interactions. Among strong interactions observed in both tumor and normal state was the interaction between proteases C1r and C1s. In fact both of these proteases form part of a complex with C1q, to make the classical pathway of the eukaryotic complement system. In serum, C1r and C1s were also found to be associated only with C1q [39]. This also indicates that the classical pathway of the complement system may still be operative in tumor cells, which tend to evade the immune system. Another important finding of this analysis was a strong association observed between beta catenin (CTNNB1) and UBC ubiquitin protein. Cytosolic beta-catenin regulates cell-to-cell adhesion however, in nucleus it acts as a component of the Wnt signaling pathway [40]. The beta-catenin signaling pathway regulates cell proliferation and is often over-expressed in cancer. Under normal conditions in the absence of wnt ligand, beta-catenin is degraded by ubiquitin mediated degradation that explains its interaction with UBC as observed in normal prostate however, its association in cancer indicates that beta-catenin may also be efficiently degraded in prostate cancer.

Among many interactions that were inferred by DC3net, the interaction between RAD50 and TRF2 is very interesting and potent. In fact, this interaction has been experimentally validated. Immuno-fluorescence and mass spectrophotometric studies has demonstrated that RAD50 complex associates with TRF2 in the S phase of cell cycle [41,42]. This is an interesting finding as this interaction was tumor specific interaction thus raising the possibility that this interaction may promote telomere maintenance in tumor cells in order to allow tumor cells to divide indefinitely.

Similarly, a strong interaction between cyclin D1 and NCOA3 a coactivator of steroid receptor coactivators (SRC) family was found. It has been demonstrated that cyclin D1 interacts with SRC-1 family coactivators in a ligand-independent fashion through a motif that resembles the leucine-rich coactivator binding motif of nuclear hormone receptors [43]. By acting as a bridging factor between estrogen receptor (ER) and SRCs, cyclin D1 can recruit SRC-family coactivators to ER in the absence of ligand. It is possible that in normal cells cyclin D1 associates with NCOA3 and activate ER leading to enhanced ER dependent transcription necessary for the growth of estrogen responsive tissue. However, what is the physiological relevance of this interaction in prostate needs to be established. Similarly it has been shown that in the process of eukaryotic protein translation, a transcriptional termination complex that harbours Tob mediates binding with polyadenylate-binding protein PABPC1 [44].

### Gene ontology analysis

To understand the relevance and physiological significance of the identified interactions, gene ontology analyses using MetaCore from GeneGo Inc. was performed. To limit the analyses to the most significant gene interactions only top 100 interactions/edges were taken for these analyses in each case.

In *enrichment by protein function *analysis for both tumor and normal case the *transcription factors *were among the top hit with z-score for tumor = 4.661; normal = 3.912 (Table 1 and 2), indicating the housekeeping function in the life of a cell, irrespective of whether they are normal or transformed.

In geneGO processes networks, among the top 10 hits in tumor network was "proliferation positive regulation_cell growth; p = 1.18 e-3" and "apoptosis_Antiapoptosis mediated by external signals via PIK3/AKT; p = 5.55 e-3" (Additional File 4, Figure S1), indicating that the identified tumor specific gene interactions may be potentially important in driving the growth in prostate cancer. In addition, gene interactions important for *translation_Translation initiation *process were also significantly enriched (p = 1.76 e-3) in tumors highlighting the need to synthesize proteins involved in tumor growth at a faster rate. Interestingly, in the normal gene interaction dataset, "Cell cycle_G1-S growth factor regulation" was among the top 10 hits (p = 5.74 e-4) (Additional File 4, Figure S2), indicating that the normal gene interactions are involved in robust regulation of cell cycle in normal cells. Among these, *Immune response_Phagocytosis *was also significantly enriched (p = 8.68 e-3), which employs that the primary immune defence to remove any cancer cells remains operative and intact in normal cellular state and one important way by which cancer cells evade host immune response is by overcoming the ability of natural killer cells to phagocytise tumor cells.

Among the top 10 *ontology-molecular functions *between tumor and normal "fructose 1,6 bisphosphate 1-phosphatase activity" (p = 8.53 e-5) and "fructose 2,6 bisphosphate 2-phosphatase activity" (p = 5.5 e-5) respectively, showed a significant enrichment (Additional File 4, Figure S3 & S4). It is important to note that fructose 1,6 bisphosphate 1-phosphatase is a key regulatory enzyme of gluconeogenesis, it is therefore vital for cancer cells to proliferate in an accelerated manner. Fructose 6 bisphosphate 2-phosphatase, however, regulates the concentration of the key positive allosteric effector of glycolysis, fructose 2,6-bisphosphate thereby is important in the regulation of energy metabolism in normal cells. Also tumor dataset was enriched in *sequence specific DNA binding transcription factor activity *(p = 3.33 e-4) while *sequence specific DNA binding *(p = 2.2 e-6) was enriched in normal gene-interactions, indicating an enhanced requirement of activation of transcription machinery in cancer cells to meet increased proliferation rate. Among the top 10 common, both the normal and tumor gene interactions, normal vital functions important for cell growth and survival such as "peptide receptor activity, G-protein coupled" (p = 2.37 e-4) was enriched (Additional File 4, Figure S5).

Analysis of *GO processes *in normal dataset resulted in *regulation of transport*; (p = 1.29 e-17), *regulation of localization*; (p = 1.9 e-17), *regulation of secretion*; (p = 5.95 e-17) and *cell-cell signalling*; (p = 2.57 e-15), as the most significant processes underscoring the important of these processes in normal cellular functioning (Additional File 4, Figure S6). In tumor gene interactions, however, two of the most significant hits among top 10 were *response to hormone stimulus*; (p = 2.35 e-13) and *response to endogenous stimulus*; (p = 3.84 e-13) (Additional File 4, Figure S7). Since prostate cancer is a well-known hormone responsive tumor, the unbiased identification of this GO process validates the relevance of these gene interaction networks identified by DC3net. This not only correlates to this gene network with disease state but also identifies potential important gene interaction in prostate cancer datasets; many of these gene interactions may be important in regulating normal cell growth and also in driving prostate cancer.

### The highest connected subnetwork of tumor differential network

**Figure 3:**
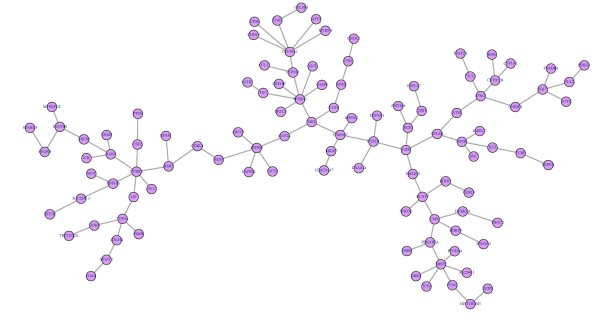
**The highest connected subnetwork in tumor difnet**. As the highest connected subnetwork with 105 edges in tumor *difnet *might have an important role in the prostate tumor cell.

Here we discuss about the highest connected subnetwork of tumor *difnet *as seen in Figure 3. This subnetwork might have an important role in prostate cancer. In fact, we found that this network demonstrates a very strong correlation among others; with the "Proliferation_positive regulation cell proliferation" which further validates that genes in this network may serve in the process of oncogenesis [45,46] (Additional File 4, Figure S8). In addition, among GO molecular functions, "growth factor activity" was found to be significantly activated indicating, that this network may stimulate growth of cancer cells (Additional File 4, Figure S9). This was further advocated by the GO processes where "Enzyme linked receptor protein signalling" pathway was among the top hits indicating the growth factors are operative via receptor kinase functions to promote the process of oncogenesis (Additional File 4, Figure S10).

## Conclusions

We showed the usage of C3NET on differential network analysis by developing the approach DC3net. The performance of it has been studied on synthetic and also on real dataset and found some verification from the literature. Since the performance of C3NET was well studied and shown to have consistently better performance than its competitors, the literature validations of the predictions and also synthetic analysis suggest that DC3net is a promising candidate to infer disease networks of direct physical gene interactions. It may be applied to any expression datasets of multiple conditions to spot the interactions of specific and also common cases. This would allow dealing with much smaller and important networks than a whole of a predicted network that mostly appears like a hairball in which case it would be discouragingly hard to find an interesting interaction for the specific problem. Therefore DC3net helps to save resources and allows spotting better drug targets to cure a disease. A future work would be considering into account transcription factor binding and sequence data along with DC3net for more specific and boosted results.

## Methods

### DC3net elaborated

**Figure 4:**
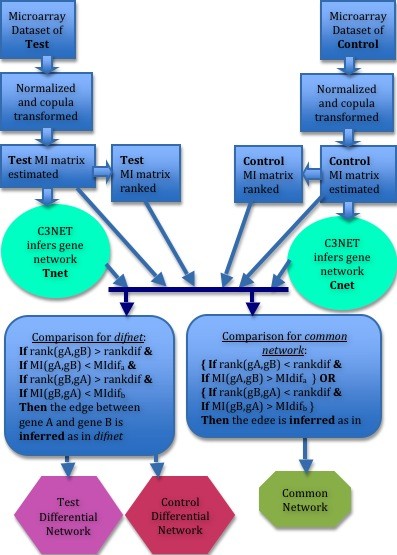
**DC3net in detail**. DC3net is elaborated by illustrating all the processes involved.

Here we describe our differential network approach with all its details over Figure 4. A general outline of DC3net was described previously. The two data sets of different conditions are first preprocessed with RMA [47] normalization and copula transformation [8,48] as in [1]. The ideal case for comparison of the two data sets is when their gene or probe names are exactly the same and their number of samples is equal or close to each other. If the gene names are not the same, e.g. test and control data sets have also different genes in the set, then it is up to user how to proceed. In any case, DC3net gives the differential network whereas the different genes will automatically appear in the *difnets *if they have significant MI values. Depends on the experimental design of microarray dataset both cases might be suitable. If the different genes is not useful to see in the differential network then at the beginning of preprocessing step all the different genes can be eliminated and then the comparison is performed with the common genes that are available in both of the networks. Or they may be at least be highlighted in the resultant network for considering the problem.

Regarding the number of samples, again the ideal case is having exactly the same samples in both datasets. However, it is usually not the case in the available biological datasets. If the datasets are from patients, namely steady state, of two different conditions (e.g. tumor and healthy) the only considerations is the number of samples in both conditions. In this case the closer the number of samples the higher the accuracy of *difnets *and *common network *because the inference performance of C3NET varies with the sample size as in the case of all other gene network inference algorithms [1,6,49]. If the data sets consists of time-series samples of two different conditions then one needs to consider that time points as well along with the number of samples. Because two different time intervals of two different conditions might result *difnets *that incorporate not only the difference in two conditions but also the differences of time positions and intervals. Therefore we can say that this analysis is more sensitive while applying DC3net to time-series data sets and one should be vigilant while interpreting the results.

We then estimate the MI matrices using the parametric Gaussian estimator as described in [50,51] and used in [1] where the details can be found. These MI matrices are square adjacency matrices where the correlation measure, MI, correspond to weight of interaction for each gene pair and the diagonals are set to zero ignoring the self-interactions. The higher the MI value the more likely that the gene pair is interacting. All gene pairs would have a MI value but some are apparently because of noise and thus nonsignificant. We also compute row wise ranked versions of these MI matrices in descending order as rank 1 corresponds to the highest MI value in a row of the matrix. This ranked matrices will be used in comparing and filtering the networks at the *comparison step*.

Then C3NET is applied to these MI matrices as explained in section *C3NET *of Additional File 1 to infer gene networks of direct physical interactions of test and control datasets independently, where the inferred networks are called as *Tnet *and *Cnet *respectively. At this point we have all the components to compare the two networks to find differential networks, *difnets*, and *common network*. We now start describing the core part of DC3net, which is the *comparison *step, as can be followed in Figure 4.

Although we describe inferring the test *difnet *in Figure 4, the same process can also be applied by considering *Cnet *and *Tnet *in place of each other to find control *difnet*. The following decision process is performed for each gene pair of *Tnet*: As seen in the *comparison *part of Figure 4, there are four conditions that all must be provided at the same time for an edge to be decided in test *difnet*. Lets say we will decide whether one of the interactions of *Tnet*, e.g. gene A (*gA*) with gene B (*gB*), only appears in *Tnet *but not in control case. We first check whether the potential interaction *gA *to *gB *of the ranked *control *MI matrix is one of top ranked interactions or not. If the rank of *gA *and *gB *in the row of *gA *of the ranked *control *MI matrix is greater than the predefined cut-off parameter, *rankdif*, then the first condition holds for deciding it as a *difnet *interaction. In our case study we set *rankdif *as 2000 since there are about 12000 genes in total in the dataset and we consider that the rank decrease to 2000 is reasonably high to be strict in the decision process. If one wants a stricter *difnet *then *rankdif *needs to be increased for example to 8000 in our case. One may also reduce *rankdif*, for example to around 200 and get more different interactions but with loose *difnet *with more edges in it. We then look at the second condition that is the change in MI value of the interaction from *gA *to *gB *in the *control *MI matrix. If the MI value of the gene pair *gA *and *gB *in the control MI matrix is less than the predefined cut-off parameter *MIdif *then second condition also holds to decide the interaction as in *difnet. MIdif *in this example is defined as *MIrate *times the maximum MI value of the row of *gA *in the *control *MI matrix. Here *MIrate *is defined as the rate decrease that is specified by the user and in our case study we set it as 0.6. Depends on how strict the differential network is desired for inferring, one may increase or decrease this cut-off parameter. The previous two conditions compared the values of the interaction from *gA *to *gB *but we also need to compare the values from *gB *to *gA *as the edge would be inferred from either of the genes since MI is a bidirectional measure. In the third condition, if the rank of the interaction from *gB *to *gA *in the row of gB in the ranked *control *MI matrix is greater than the cut-off parameter, *rankdif*, then the third condition also holds. For the fourth condition, if the MI value of the interaction from *gB *to *gA *in the *control *MI matrix is less than the parameter *MIdif *then the fourth condition also holds. Note that here, *MIdif *is set with respect to the row of *gB *in the *control *MI matrix and thus may be denoted as *MIdif_b _*(the previous one of the condition two could be denoted as *MIdif_a_*). In this particular example of *gA *and *gB*, if four of the conditions hold together then we infer this interaction as in *test difnet *and continue to perform same filtering process for all other gene pairs in *Tnet*. In the end, we get *test difnet *as a smaller subnetwork of *Tnet*. This was the description of inferring test *difnet *(or tumor *difnet *in our case study). However, inferring the control or normal *difnet *can be performed by applying the same procedure described for *Tnet *after interchanging the place of *Cnet *and *Tnet *in the above descriptions. We now continue by describing the inference of the *common network*.

Lets now start to describe the way we infer the *common network*. The strictest way of finding the common network is to search for all the same interactions between *Tnet *and *Cnet *and then infer the *common network*. On the other hand one may choose not to be that strict and consider also their ranks and MI value decreases in the other dataset. More precisely, one needs to follow the manner of the *difnet *process described above but change the comparison operator of rank difference, *rankdif*, from greater to less and for the *MIdif *from less to greater as can be seen in Figure 4. Furthermore, we only look at either of the two conditions, rather than all the four conditions together, from *gA *to *gB *or *gB *to *gA*. The logical OR operator in Figure 4 represents this process. Then following the *difnet *process with the changes described above will result the *common network*. Again the cut-off parameters need to be arranged by the user regarding the biological datasets dealt with. One may also assign different cut-off parameter values for *difnet *and *common network *inference to place a gap in the inferred networks for more accurate results by avoiding decision around the cut-off boundaries. Moreover, one may also choose to use only one of the cut-off parameters (either rank or MI value) for looser or maybe stricter cases. In our case study on the prostate cancer data, we set different parameters such that *rankdif *as 200 and *MIdif *as 0.85 for more strict results while inferring the *common network*. Note that although we described the approach DC3net for the analysis of two different cell conditions, it is straightforward to extend it to multiple conditions in a similar fashion. It is worth mentioning that, in its current form, we infer the *common network *with respect to the common interactions of *tumor difnet *comparing to *control *(normal) MI matrix, since our focus is the disease case. Nevertheless, if desired, a more general *common network *can also be inferred by adding one more step to the inference of it as described above. In that case, interactions of *Cnet *is compared to the tumor (test) MI matrix in the same way as performed for the test case and then the filtered interactions added onto the *common network*.

We provide some more details on C3NET and the application of DC3net over a synthetic dataset in Additional File 1 where as we give the related references [52-55] of them in this main paper.

On the other hand, it is always beneficial to mention about the limitations of any algorithm for users. One of the main limitations of any differential network analysis method, including DC3net, is that they need similar number of samples of multiple conditions for the most accurate comparison possible. It is because the inference performances of GNI methods, including C3NET, are affected by sample sizes. DC3net may still be applied on expression datasets with different sample sizes as long as one keeps in mind the fact that the results might have been biased by the difference in sample sizes. Another limitation is that microarray experiments do not always provide the exact same probes as some of them may be distorted during experiment. Therefore, because of the imprecision of available the datasets, some, though few, genes may not be compared with the other condition. Those genes cannot be considered condition specific and needs further attention. The general limitations of inference algorithms apply on our differential analysis. Particularly, DC3net inherently deals with only the very core, but not all, of the true underlying network because of the conservative causal core property of C3NET but in return gains in prediction accuracy.

### The biological dataset

In order to perform a real application of the presented approach DC3net, we used the dataset of [56] that was used to study clinical prostate cancer behaviour. This dataset is widely used in the literature and also quite suitable for differential network analysis as it has almost equal number of samples for tumor and normal cases. The data were gathered by analysing 235 radical prostatectomy specimens from patients undergoing surgery between 1995 and 1997. It was reported that 65 of these samples had tumors on opposing sides of the tissue specimen. High-quality expression profiles were derived from 52 of these prostate tumors and 50 nontumor (e.g. normal) prostate samples with oligonucleotide microarrays containing probes for approximately 12,600 genes and ESTs [56]. The raw data has been downloaded from http://www-genome.wi.mit.edu/MPR/prostate. As the number of samples in tumor and normal are close, we are able avoid the sample size effect differences on the inference of both datasets.

## Competing interests

The authors declare that they have no competing interests.

## Authors' contributions

GA has conceived the study, designed DC3net, performed the analysis and wrote the manuscript. MA performed biological analysis and wrote the related text. FM and DN supervised and coordinated the study. All authors read and approved the final manuscript.

## Supplementary Material

Additional file 1 **Details of synthetic dataset analysis and C3NET**. Click here for file

Additional file 2 **All the inferred differential and common networks**. All the inferred interactions of *difnets *and common network in tab-delimited format. (.xls) with MI edge weight and rank information. Click here for file

Additional file 3 **Plots of the inferred differential and common networks**. A *pdf *file consisting of the plots for interesting subnetworks of the *difnets *and *common network*. It also includes all the subnetworks of these networks with oncogenes. Click here for file

Additional file 4 **Enrichment analysis illustrations using MetaCore from GeneGo Inc**.. Each figure in the file is referred in the main text. Click here for file
