# Supplementary material for: Differential C3NET reveals disease networks of direct physical interactions
Source: BMC Bioinformatics. 2011 Jul 21;12:296. doi: 10.1186/1471-2105-12-296 (PMC3156794; doi:10.1186/1471-2105-12-296)
Supplement: Additional file 1 — Details of synthetic dataset analysis and C3NET. [file 1471-2105-12-296-S1.PDF]

Additional File 1 for “Differential C3NET reveals disease networks of direct physical interactions”

Gökmen Altay[1§], Mohammad Asim[1,2], Florian Markowetz[1,2], David E. Neal[1,2]

[1]Department of Oncology, University of Cambridge, Cambridge Research Institute, CB2 0RE, Cambridge, UK

[2]Cancer Research UK Cambridge Research Institute, Li Ka Shing Centre, CB2 0RE, Cambridge, UK

[§]Corresponding author

Email addresses:

GA: ga303@cam.ac.uk

MA: mohammad.asim@cancer.org.uk

FM: florian.markowetz@cancer.org.uk

DN: den22@cam.ac.uk

Abstract

We have expressed some of the supportive parts of the study in this Additional File. We express, in detail, the application of DC3net on a synthetic dataset and also overview of C3NET here. The references given here can be seen in the main manuscript.

Methods

C3NET

A detailed explanation and example of the C3NET can be found in [1]. In order not to reproduce the open access text, we briefly describe the GNI algorithm C3NET and the way we applied it to our datasets. As explained in [1], we first preprocess the raw microarray data using RMA [46] normalization and apply copula transform [47, 1] to the normalized data and then C3NET is applied to it to infer gene network of direct physical interactions from the dataset. C3NET has two basic steps. At the first step, using information theory based correlation measure entropy, MI matrix is generated. It is a square matrix that includes MI weights for each pair of genes and its diagonal is set to zero as self-interactions are not taken into account. Then non-significant MI weights are eliminated via a resampling test with which an arbitrary p-value or a multiple testing correction, such as false discovery rate (FDR), might be used. As the resampling tests results MI thresholds usually below mean of MI matrix, we have set the cut-off threshold to upper quartile of the upper triangle of the MI matrix in both conditions to get more strict MI values with the quickest time. Then the second step of C3NET is applied to this MI matrix of significant MI values. For each gene, only one interaction is inferred, which corresponds to the maximum MI value for that gene.

Namely, the strongest link for each gene is inferred with C3NET. This is the most conservative inference method and thus is very useful in differential network analysis. Further details of C3NET with examples can be found in [1]. C3NET showed consistently better inference performances over various network topologies, as it only aims to infer just the core of the gene network rather than the whole set of interactions. However we might estimate that C3NET is expected to infer approximately 1/3 of the whole gene network as it was reported that majority of genes have few interactions, whereas a small number are highly connected and serve as network hubs [51].

Synthetic dataset analysis

When a method is developed, it is always useful to apply initially on synthetic datasets that are artificially produced from known networks, which in turn allows comparing the predicted networks with the reference networks. Although it is difficult to estimate how good is the simulation with respect to the implementation on real biological datasets, results give an idea of the best-case scenario. If the performances are not promising in synthetic simulations then there is no reason at all to try a method on real datasets. Having this motivation in mind, we produced a benchmark network by randomly generating a subnetwork from a published real *E.coli* network [52, 53]. It consists of 400 genes with 864 interactions and assumed to be the gene network of healthy cell and taken as reference *control network* in the simulations. In this network, we have changed the place of 8 of interactions arbitrarily and obtained a second network and assumed it as the gene network of tumor cell and thus took it as reference *test network*. In order to generate synthetic microarray datasets of these two networks, we used the expression data simulator SynTReN [54] just like in [1]. Sample size is set to 800 in both cases and steady-state expression values including noise (the

parameter is set to 0.05) are generated. We then followed all the steps of our approach *difnet* as illustrated in Figure 4 of the main papaer. As a result, we inferred *disease network* (e.g. the different interactions of *test network* with respect to control dataset), the normal network (e.g. the different interactions of *control network* with respect to test dataset) and the *common network* (the interactions that appears in both cases). The results are extremely promising and encouraging as illustrated in the Venn diagram in Figure S1. As we have the reference network, we are able to tune the filtering parameters *rankdif* and *MIdif* to get the best performance. Although this is not the case in real implementation, we observe the values of these parameters that can then be used as a reference to estimate them in real implementation regarding the number of genes in datasets. In Figure 4, we set *rankdif* as 10 and *MIdif* as 90% for comparing *common network* and set *rankdif* as 12 and *MIdif* as 88% for *difnets*, considering the fact that the number of genes in the dataset is just 400. Each gene pair such as gene A and gene B in *Tnet* means that the MI from gene A to gene B is the highest MI of gene A and thus gene B is the top ranked (rank 1) neighbour gene of gene A. In this example, by setting *rankdif* as 10 we hypothesize that if the rank of gene A to gene B in ranked *control* MI matrix is in top 10 then we assume that this interaction is still a common interaction in both test and control cases since the rank of *control* case still quite high. If the rank was greater then we declare that this interaction gene A to gene B only appears in test case but not in control case. In order to keep a balance between rank difference and the change in MI values of an interaction in both cases we also look at the difference in MI value changes *MIdif*. This means that if the MI value of the interaction of gene A to gene B decreases to lower than 90% of the maximum MI value of gene A in *control* case, then we assume that this interaction only appears in *test* case but not *control*. Otherwise if it is greater than 90 % then we say that this is a

common interaction of both cases. Depends on the biological problem, sample size and number of genes of datasets one may choose to employ only one of these decision parameters, whereas we want to be somewhat strict on the inference and use both of them together while filtering for decision.

Using the parameters above in *difnet*, as can be followed from Figure S1, we inferred the *disease network* with 10 interactions of which 6 of them are TP (true positive). Recall that there were 8 TP available to be inferred. We also inferred the *common network* with 208 interactions of which 205 of them are TP. We then inferred *control network* with 14 interactions of which 6 of them are TP. In *Tnet* and *Cnet*, it was predicted by C3NET that they have 220 and 224 interactions, respectively. As can be clearly seen from this example that, a biologist can be more easily detect disease related interactions by analysing only 10 interactions instead of out of 220. This example shows how useful the presented approach *difnet* might be.

As an application note, in the first step of C3NET we used upper quartile value of the upper triangle of MI matrix as a significance threshold as this is already very strict cut-off for eliminating nonsignificant MI values in the MI matrix [1, 6]. For a looser cut-off we slightly loose in performance as follows. When we take the MI cut-off as mean of upper triangle of MI matrix then we have these inference values. In *Tnet* and *Cnet*, it was predicted by C3NET that they have 300 and 290 interactions, respectively. We inferred the *disease network* with 12 interactions of which 6 of them are TP. We inferred *control network* with 26 interactions of which 6 of them are TP. We also inferred the *common network* with 265 interactions of which 220 of them are TP. As can be seen, a dramatic change in the MI significance threshold caused a slight decrease in the accuracy of the results.

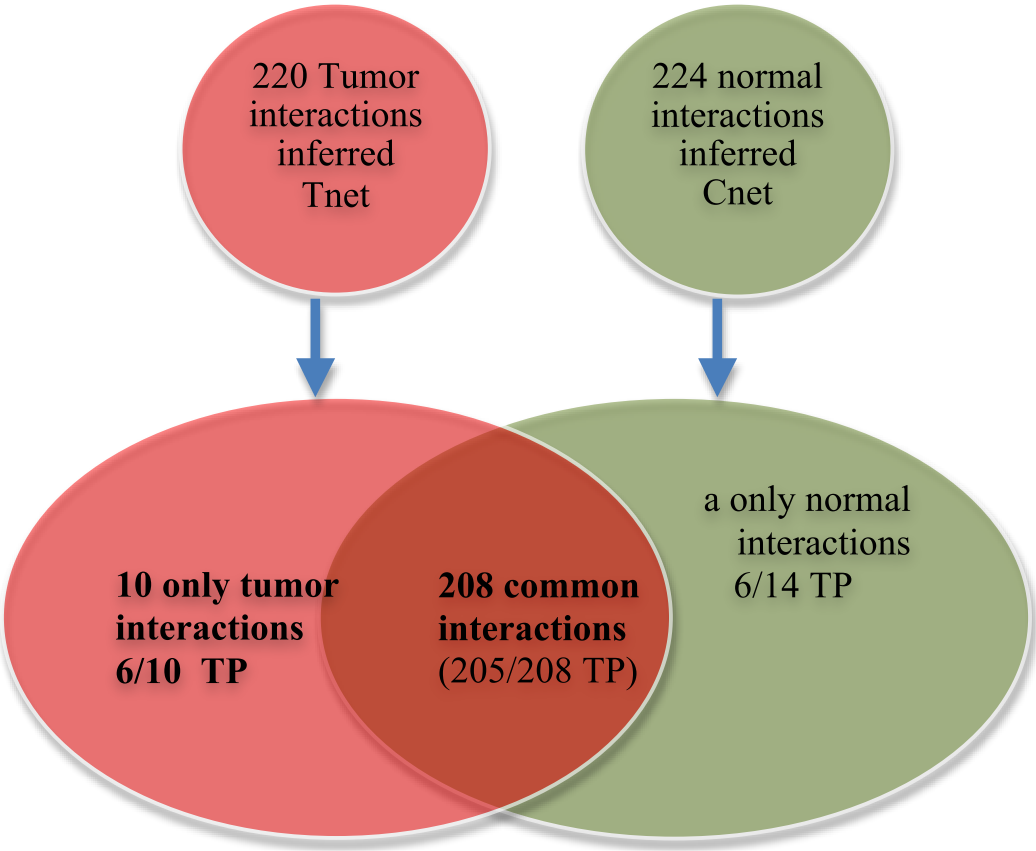

Figure S1 . Results of *difnet* on the synthetic dataset. As an example, 10 edges predicted as tumor specific and 6 of them are TP, where in reality 8 tumor specific edges exist.
