# Supplementary material for: Differential C3NET reveals disease networks of direct physical interactions
Source: BMC Bioinformatics. 2011 Jul 21;12:296. doi: 10.1186/1471-2105-12-296 (PMC3156794; doi:10.1186/1471-2105-12-296)

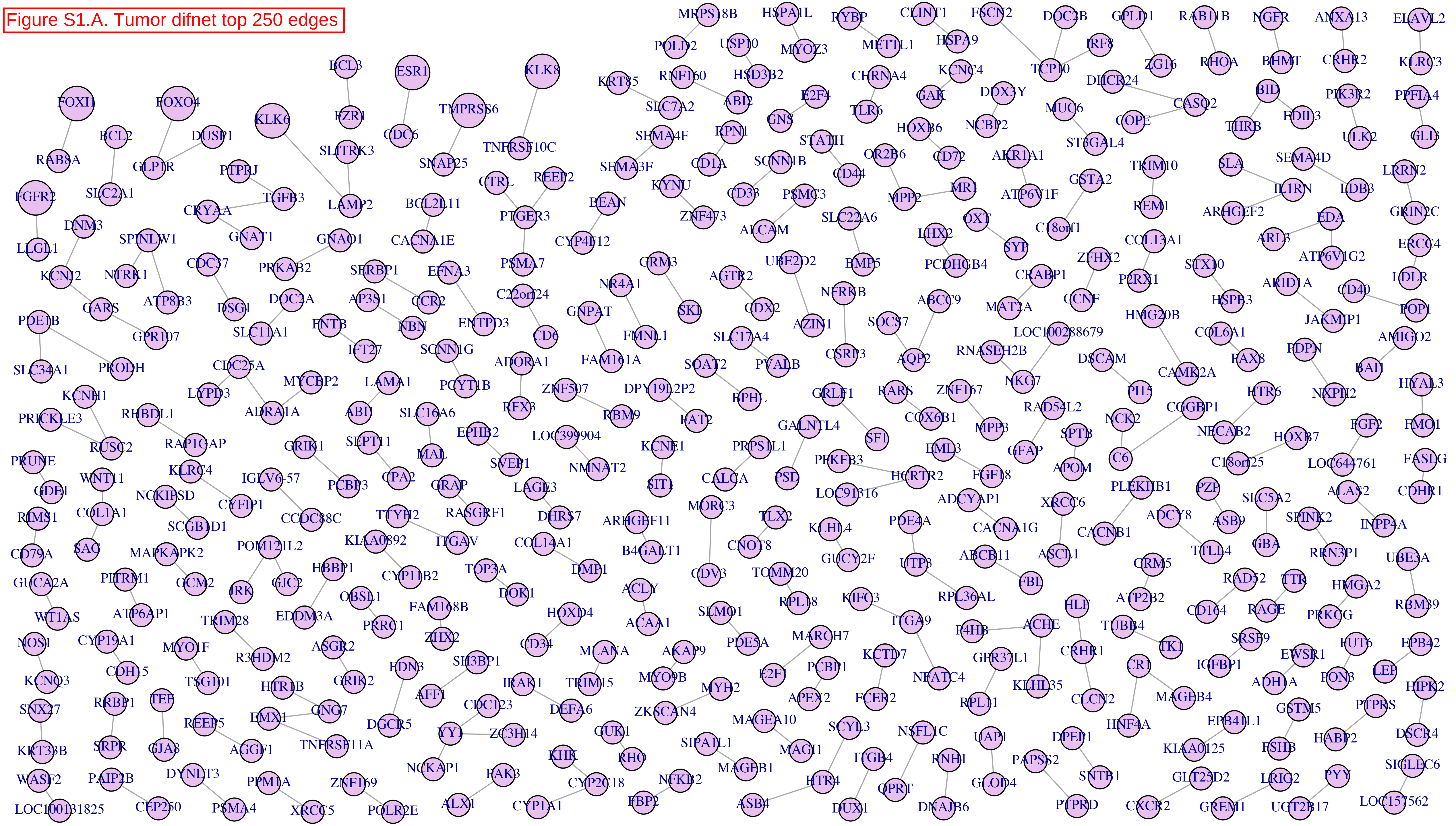

Figure S1.A. Tumor difnet top 250 edges

Figure S1.B. Tumor difnet hubs > 3

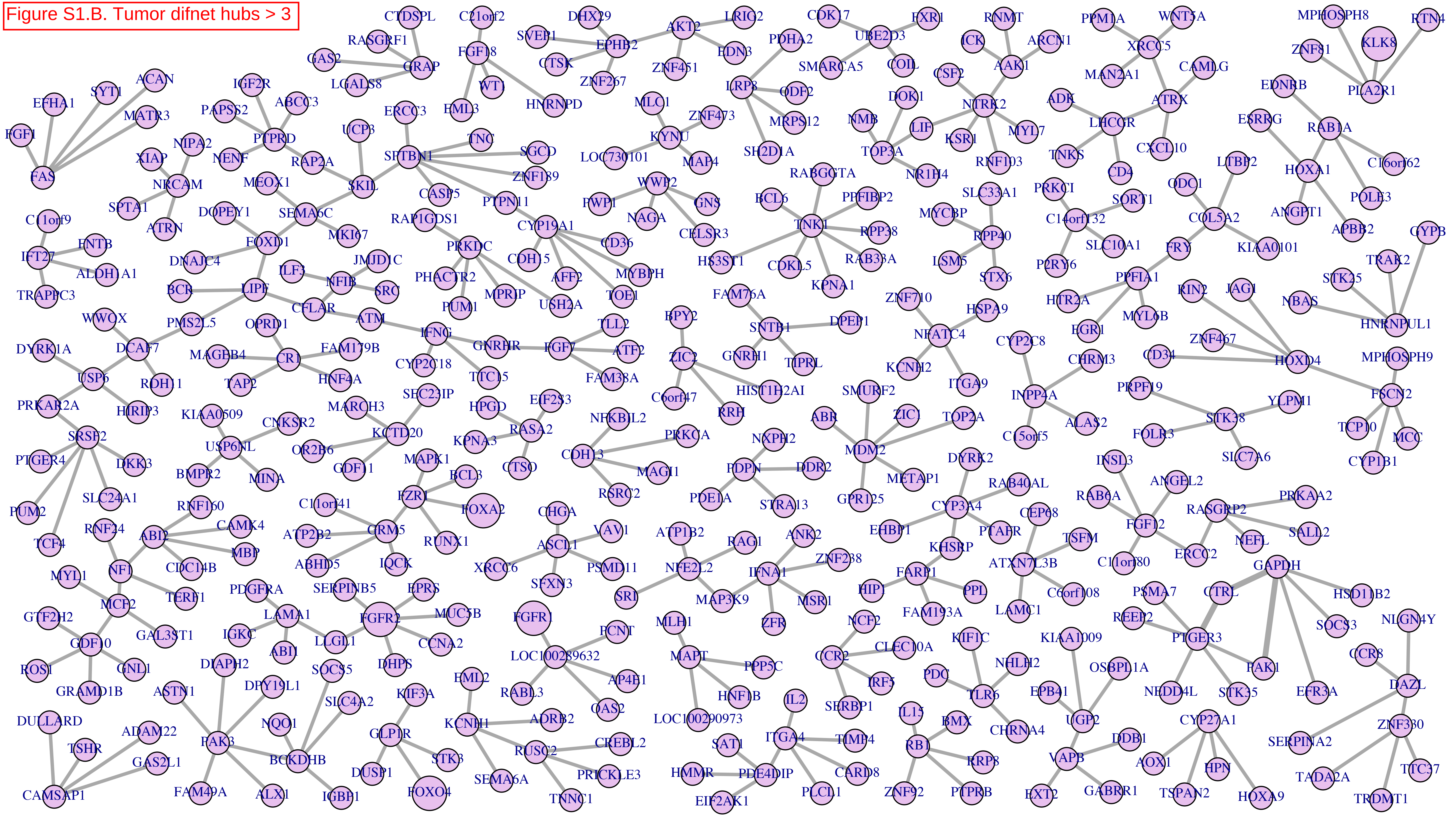

Figure S1.C Tumor difnet hubs > 2 and < 4

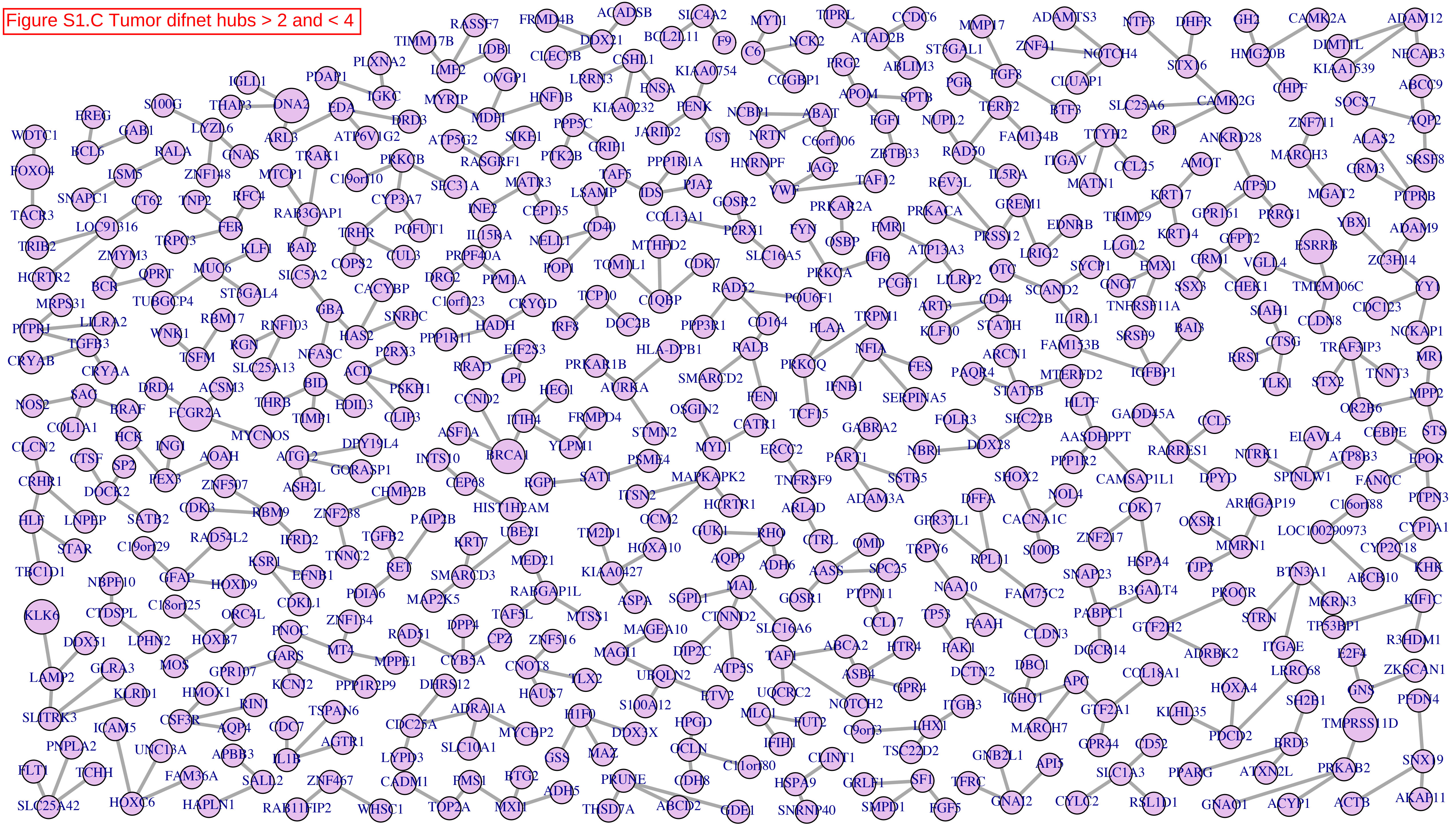

Figure S2.A. Normal difnet top 250 edges

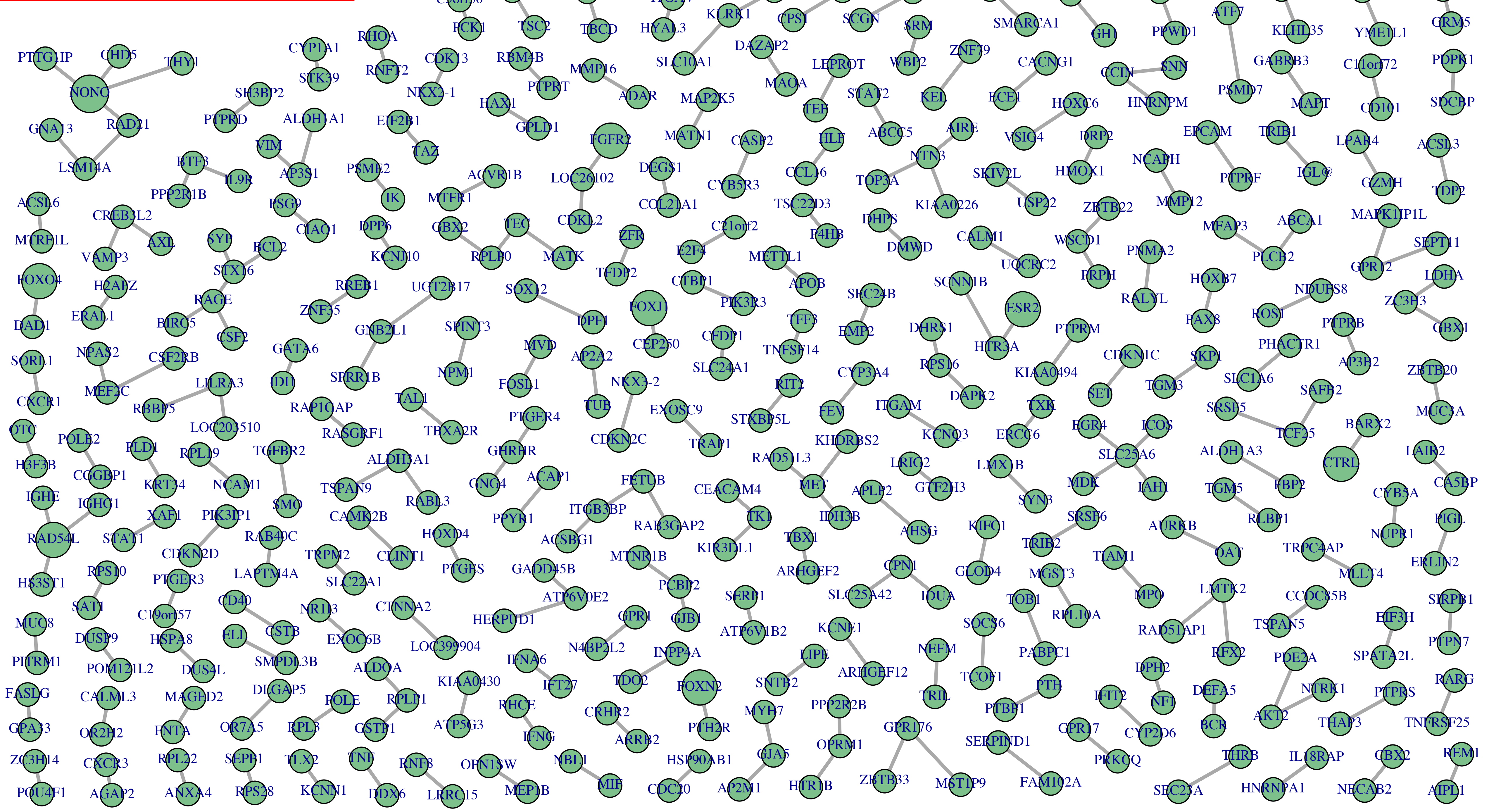

Figure S2.B. Normal difnet hubs > 3

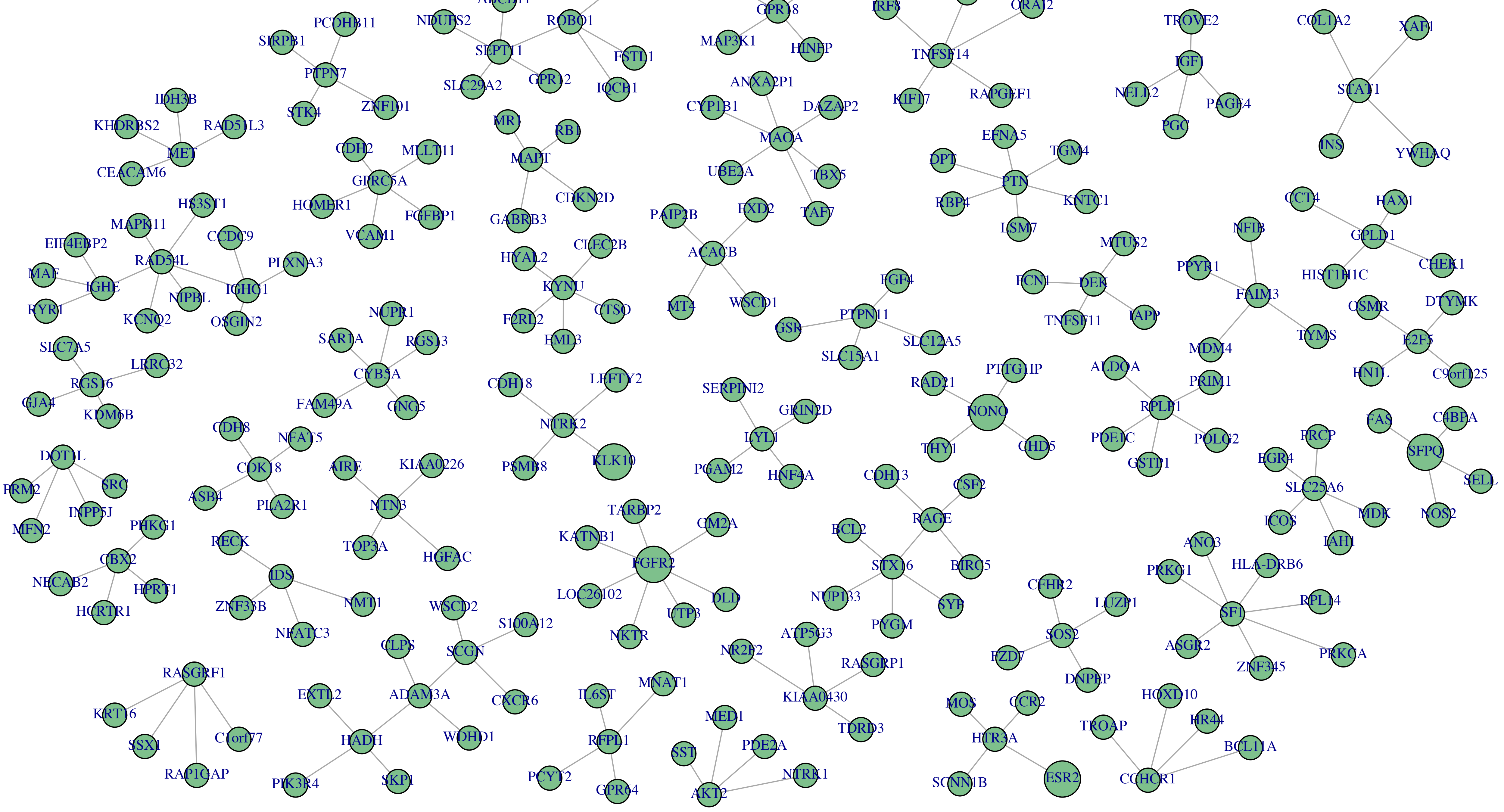

Figure S2.C. Normal difnet hubs > 2 & < 4

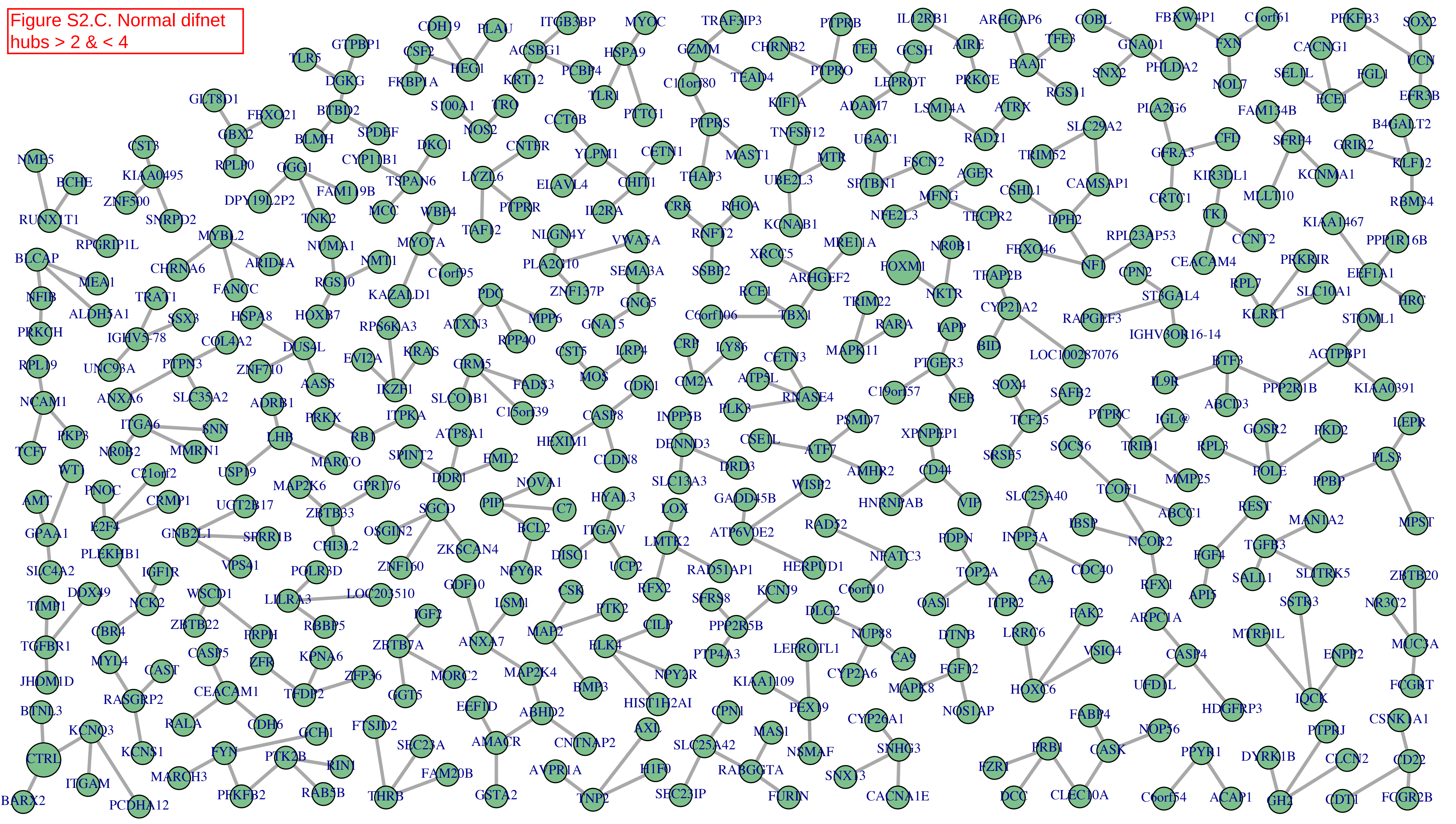

Figure S3.A. Common network of top 300 edges

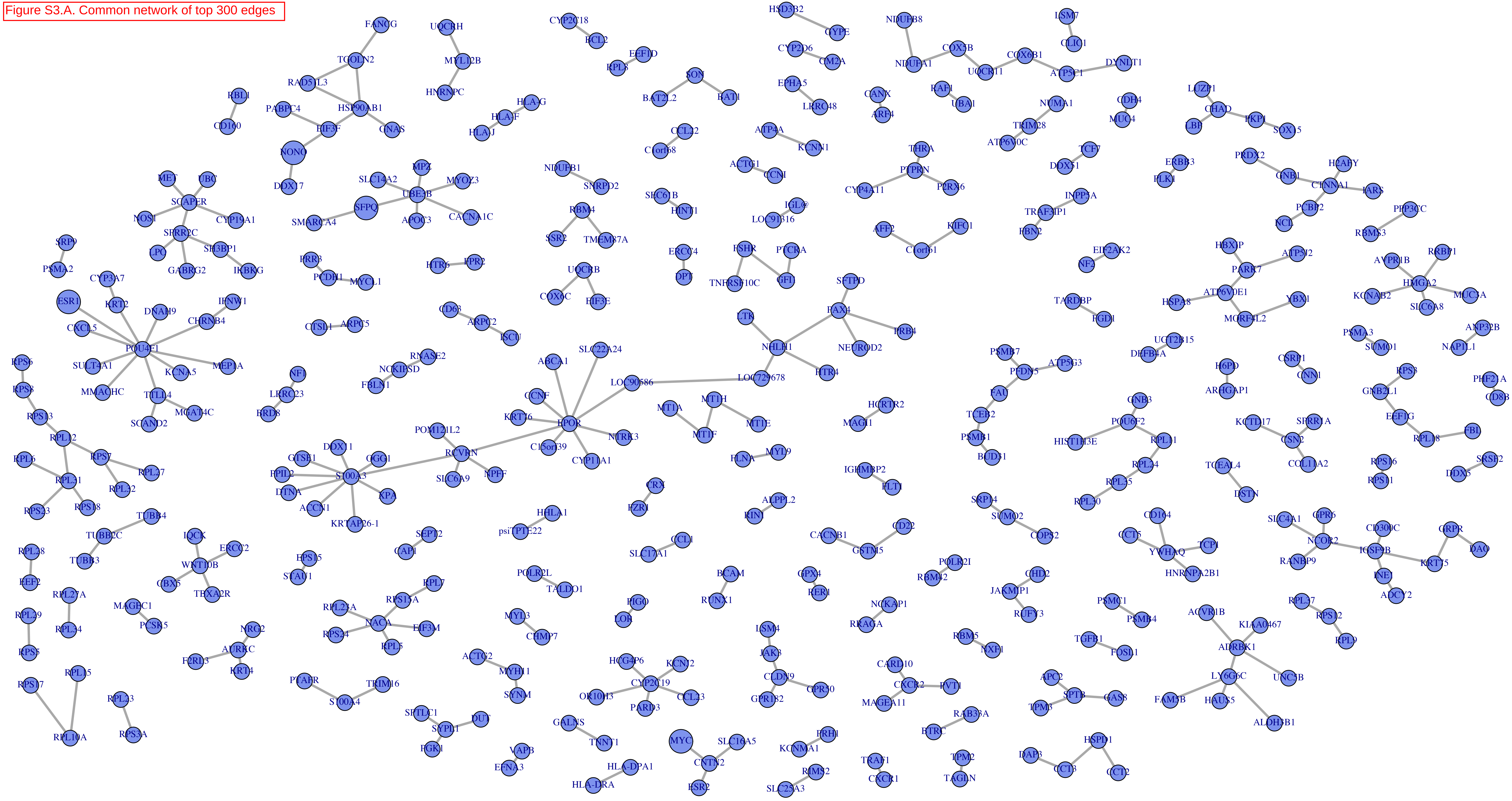

Figure S3.B. Common network of hubs > 2

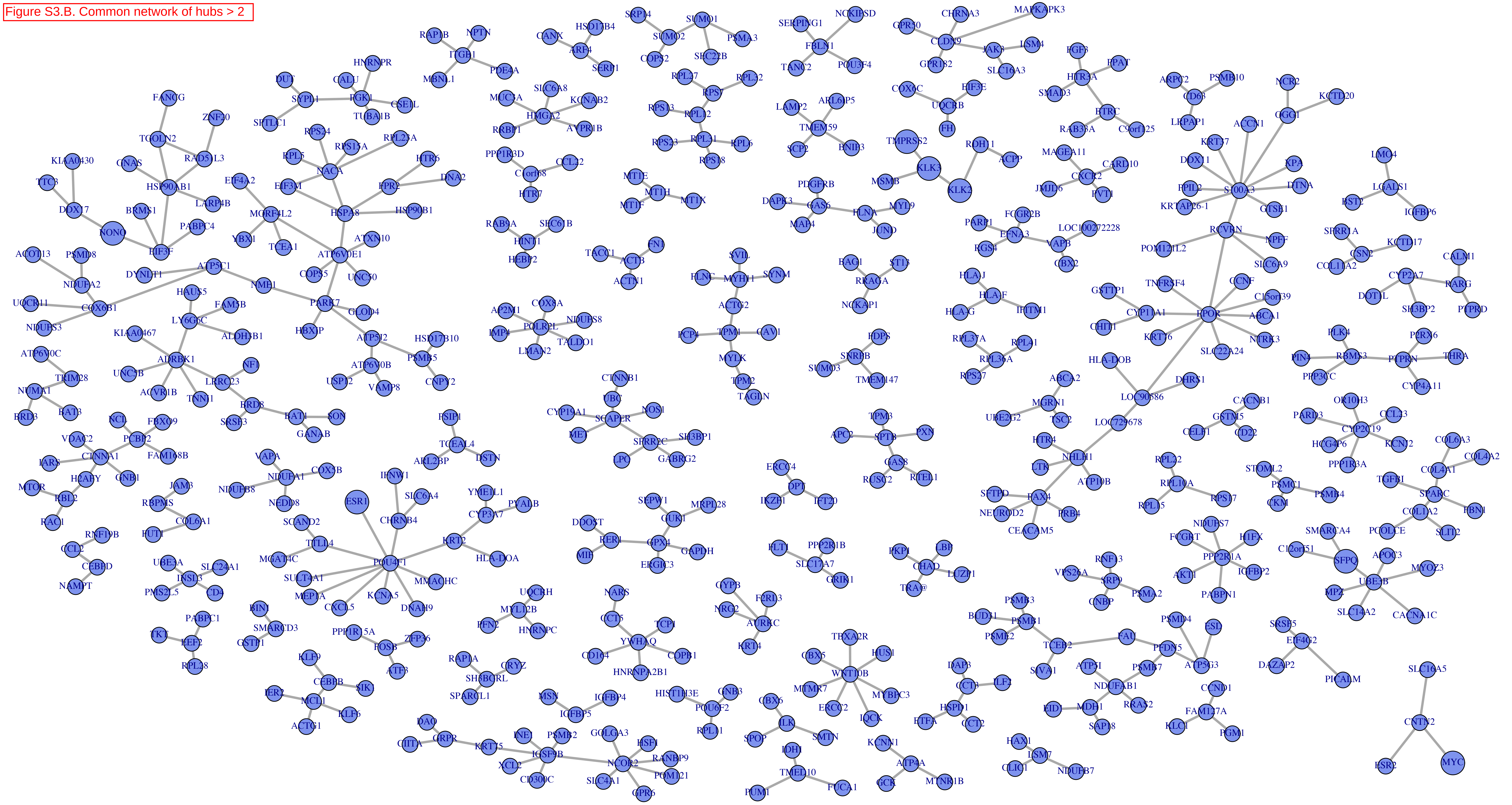

Figure S3.C. Common network exact overlapping edges

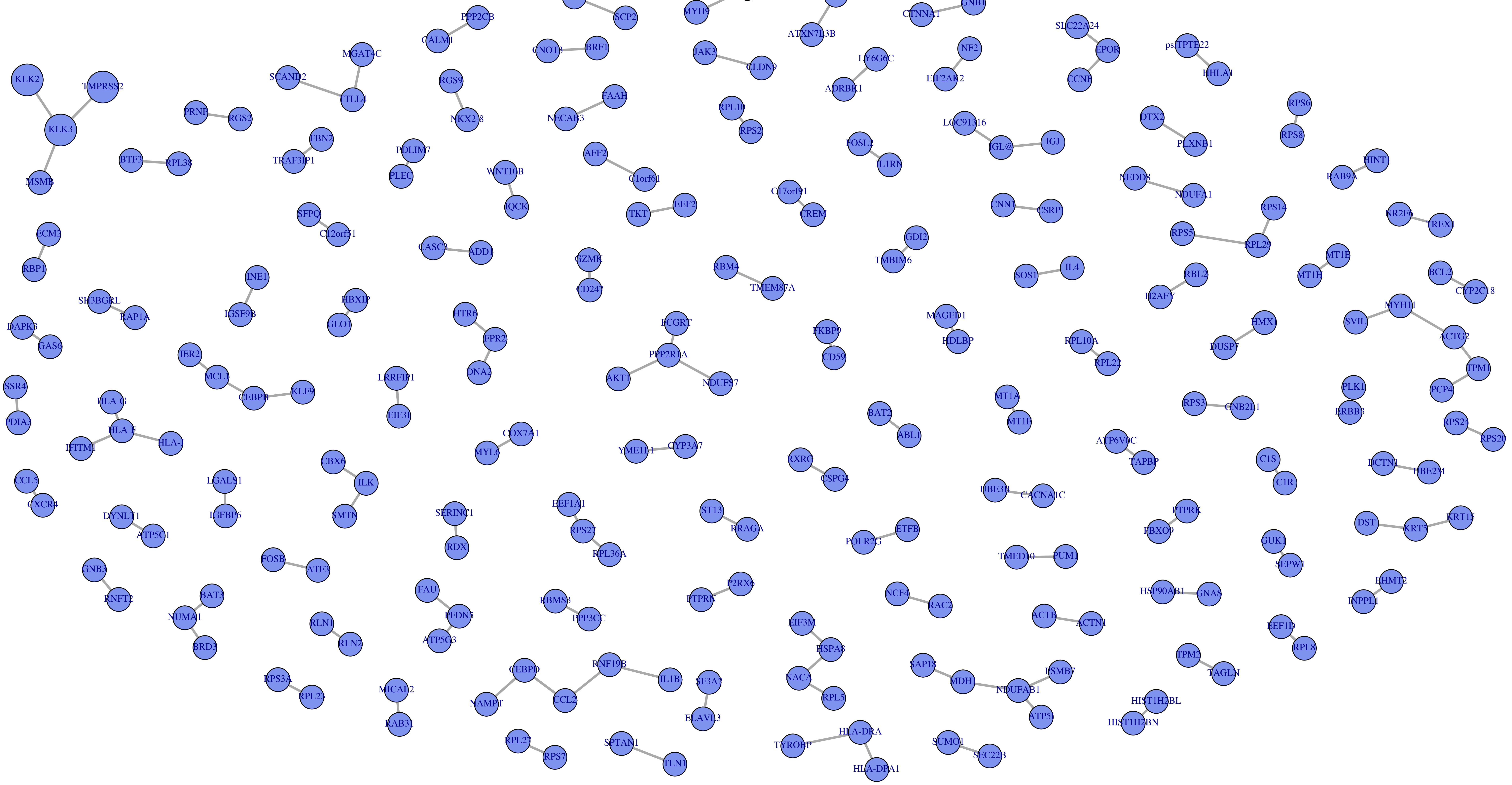

Figure S4.A. Oncogenes of tumor difnet

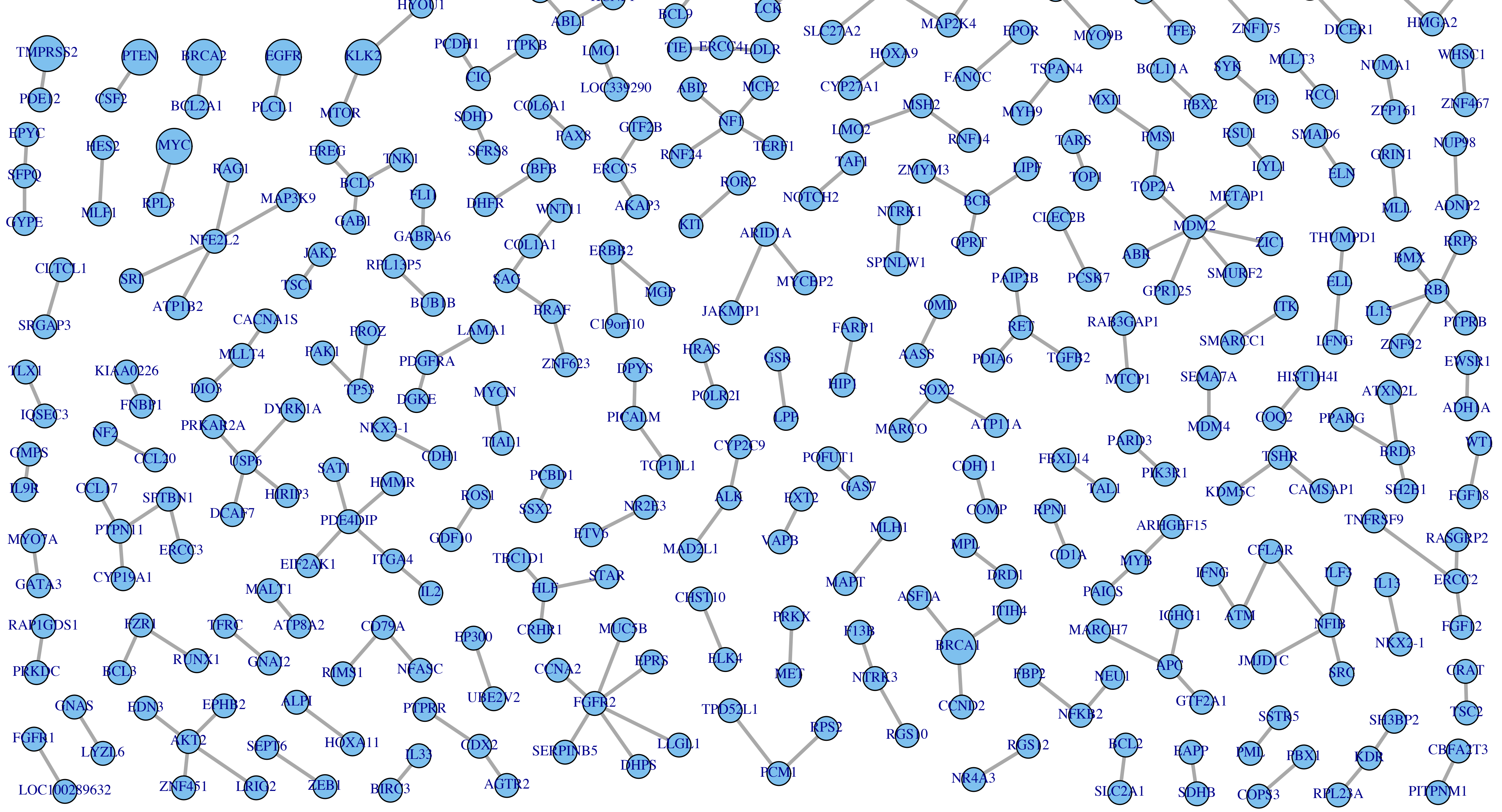

Figure S4.B. Oncogenes of normal difnet

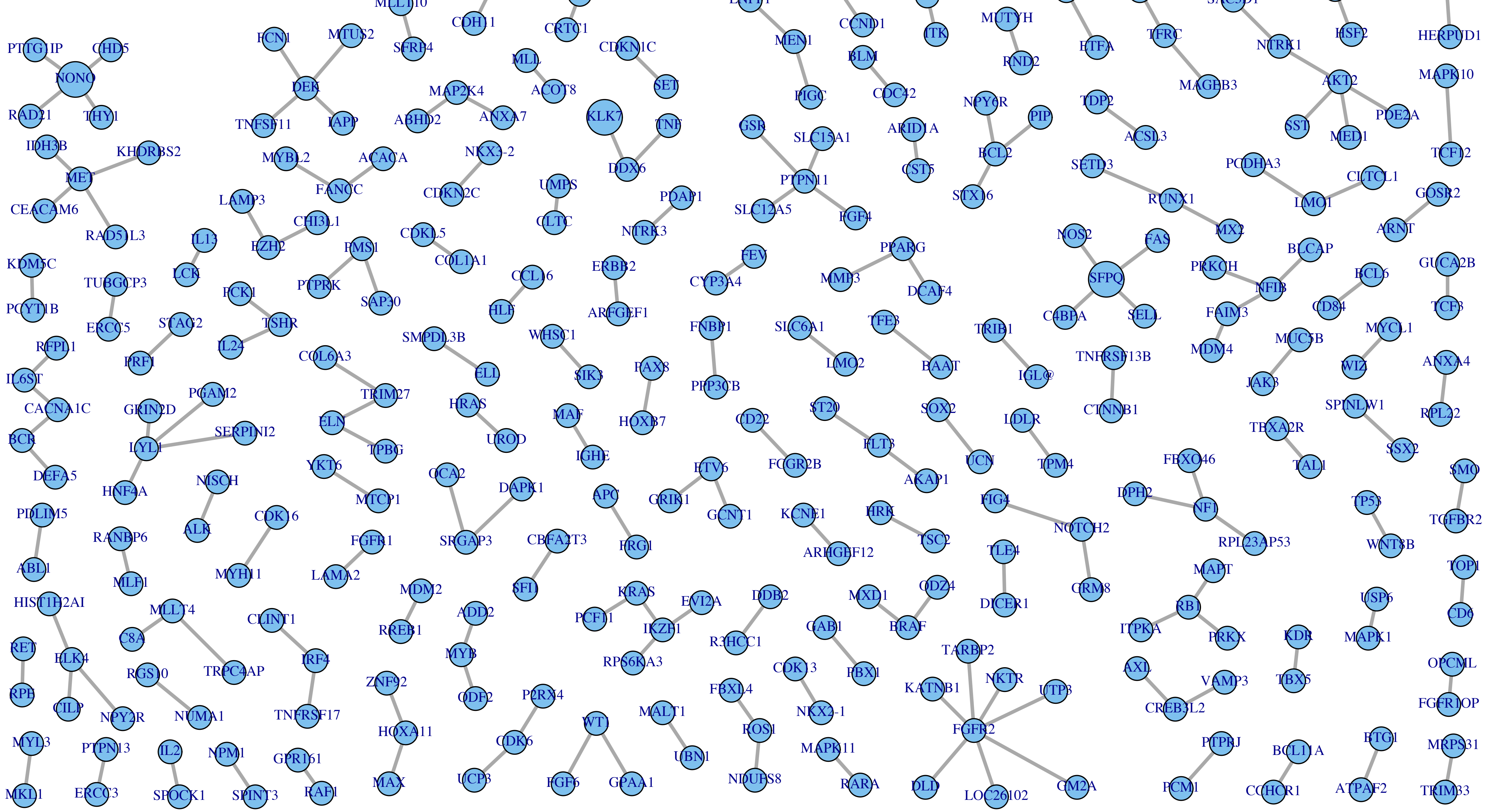

Figure S4.C. Oncogenes of common network

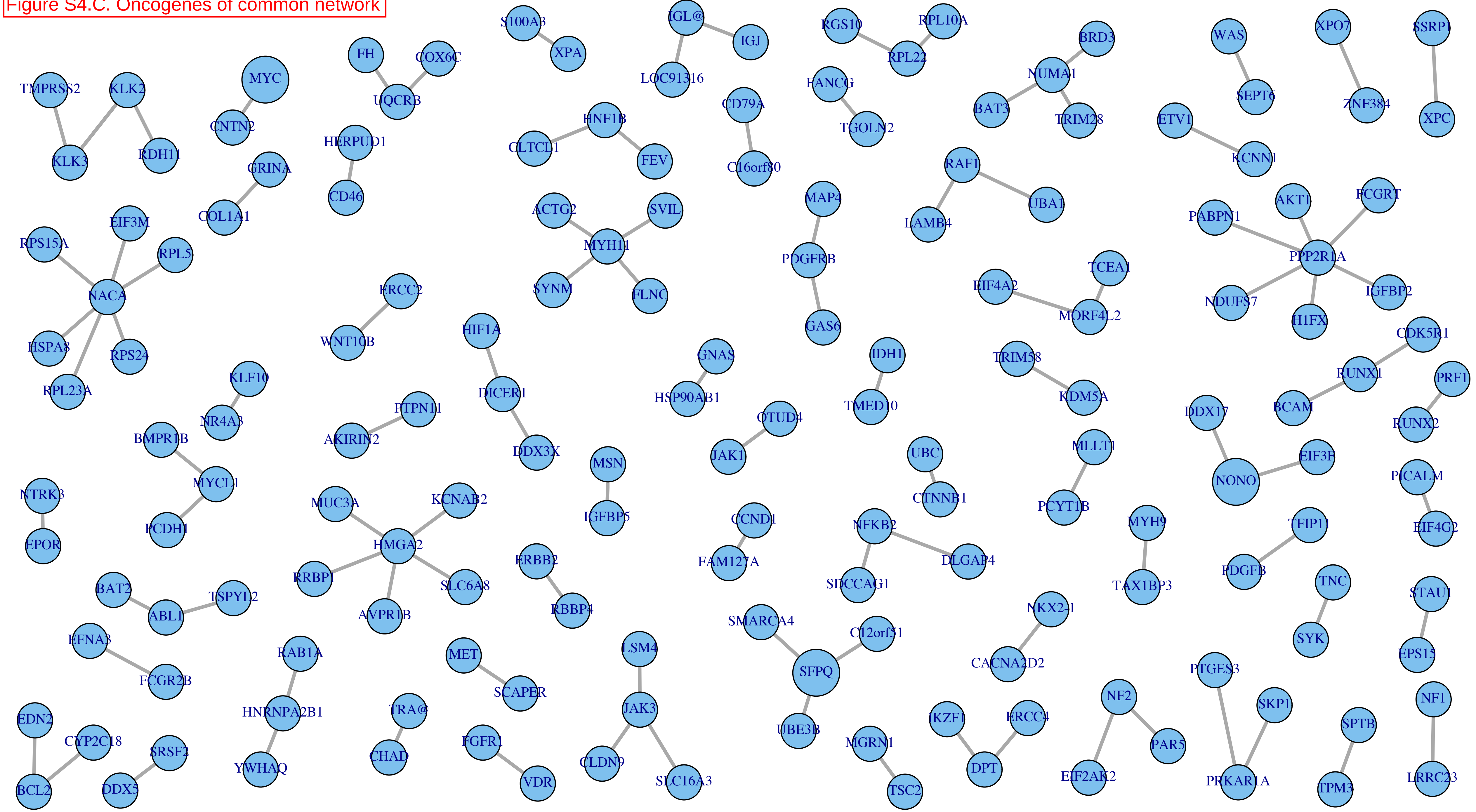

Supplement: Additional file 3 — Plots of the inferred differential and common networks. A pdf file consisting of the plots for interesting subnetworks of the difnets and common network. It also includes all the subnetworks of these networks with oncogenes. [file 1471-2105-12-296-S3.PDF]
