# Supplementary material for: Differential C3NET reveals disease networks of direct physical interactions
Source: BMC Bioinformatics. 2011 Jul 21;12:296. doi: 10.1186/1471-2105-12-296 (PMC3156794; doi:10.1186/1471-2105-12-296)
Supplement: Additional file 4 — Enrichment analysis illustrations using MetaCore from GeneGo Inc.. Each figure in the file is referred in the main text. [file 1471-2105-12-296-S4.PDF]

Figure S1

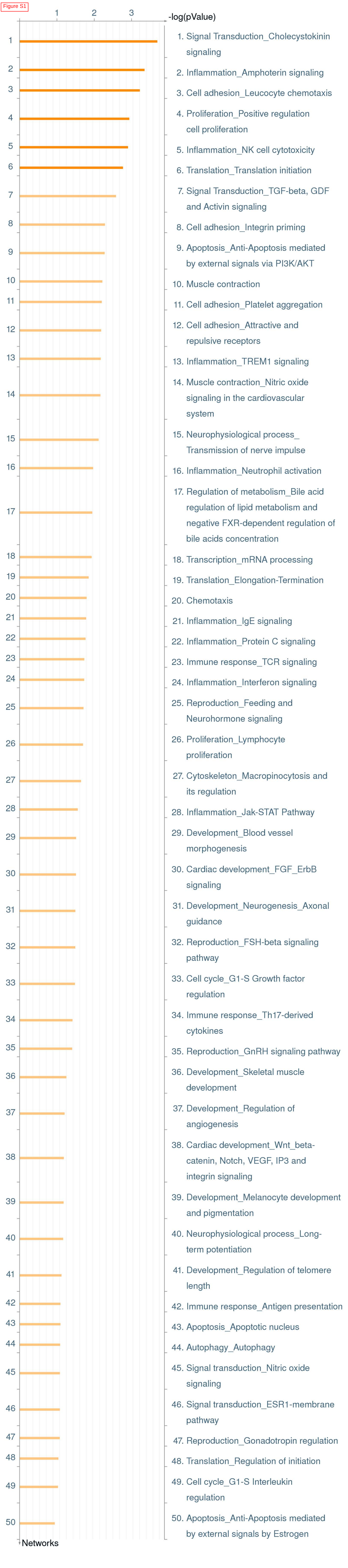

1
2
3
-log(pValue)
Networks
1. Signal Transduction_Cholecystokinin signaling
2. Inflammation_Amphoterin signaling
3. Cell adhesion_Leucocyte chemotaxis
4. Proliferation_Positive regulation cell proliferation
5. Inflammation_NK cell cytotoxicity
6. Translation_Translation initiation
7. Signal Transduction_TGF-beta, GDF and Activin signaling
8. Cell adhesion_Integrin priming
9. Apoptosis_Anti-Apoptosis mediated by external signals via PI3K/AKT
10. Muscle contraction
11. Cell adhesion_Platelet aggregation
12. Cell adhesion_Attractive and repulsive receptors
13. Inflammation_TREM1 signaling
14. Muscle contraction_Nitric oxide signaling in the cardiovascular system
15. Neurophysiological process_ Transmission of nerve impulse
16. Inflammation_Neutrophil activation
17. Regulation of metabolism_Bile acid regulation of lipid metabolism and negative FXR-dependent regulation of bile acids concentration
18. Transcription_mRNA processing
19. Translation_Elongation-Termination
20. Chemotaxis
21. Inflammation_IgE signaling
22. Inflammation_Protein C signaling
23. Immune response_TCR signaling
24. Inflammation_Interferon signaling
25. Reproduction_Feeding and Neurohormone signaling
26. Proliferation_Lymphocyte proliferation
27. Cytoskeleton_Macropinocytosis and its regulation
28. Inflammation_Jak-STAT Pathway
29. Development_Blood vessel morphogenesis
30. Cardiac development_FGF_ErbB signaling
31. Development_Neurogenesis_Axonal guidance
32. Reproduction_FSH-beta signaling pathway
33. Cell cycle_G1-S Growth factor regulation
34. Immune response_Th17-derived cytokines
35. Reproduction_GnRH signaling pathway
36. Development_Skeletal muscle development
37. Development_Regulation of angiogenesis
38. Cardiac development_Wnt_beta-catenin, Notch, VEGF, IP3 and integrin signaling
39. Development_Melanocyte development and pigmentation
40. Neurophysiological process_Long-term potentiation
41. Development_Regulation of telomere length
42. Immune response_Antigen presentation
43. Apoptosis_Apoptotic nucleus
44. Autophagy_Autophagy
45. Signal transduction_Nitric oxide signaling
46. Signal transduction_ESR1-membrane pathway
47. Reproduction_Gonadotropin regulation
48. Translation_Regulation of initiation
49. Cell cycle_G1-S Interleukin regulation
50. Apoptosis_Anti-Apoptosis mediated by external signals by Estrogen

Figure S2

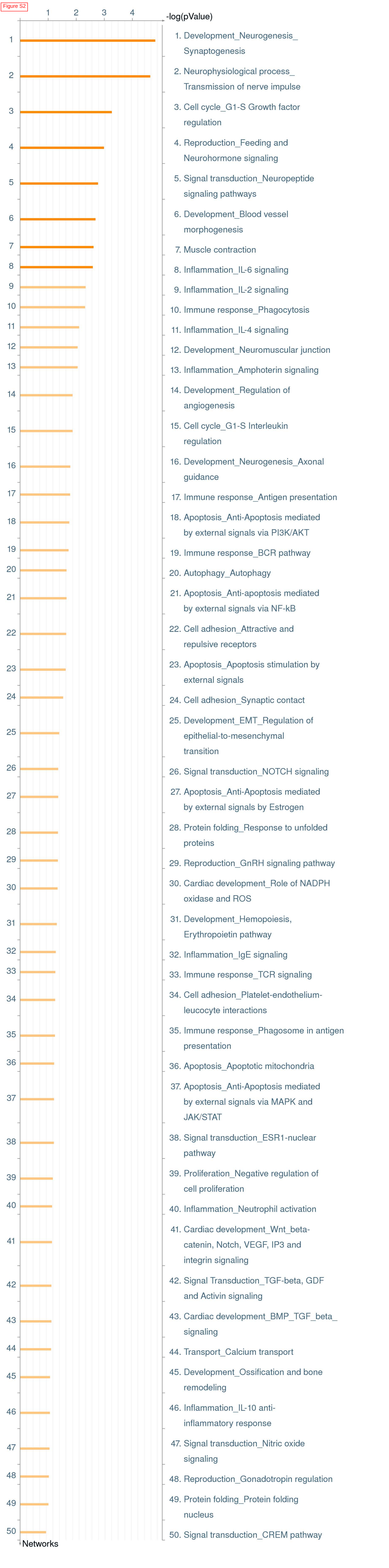

-log(pValue)
1
2
3
4
Networks
1. Development_Neurogenesis_Synaptogenesis
2. Neurophysiological process_Transmission of nerve impulse
3. Cell cycle_G1-S Growth factor regulation
4. Reproduction_Feeding and Neurohormone signaling
5. Signal transduction_Neuropeptide signaling pathways
6. Development_Blood vessel morphogenesis
7. Muscle contraction
8. Inflammation_IL-6 signaling
9. Inflammation_IL-2 signaling
10. Immune response_Phagocytosis
11. Inflammation_IL-4 signaling
12. Development_Neuromuscular junction
13. Inflammation_Amphoterin signaling
14. Development_Regulation of angiogenesis
15. Cell cycle_G1-S Interleukin regulation
16. Development_Neurogenesis_Axonal guidance
17. Immune response_Antigen presentation
18. Apoptosis_Anti-Apoptosis mediated by external signals via PI3K/AKT
19. Immune response_BCR pathway
20. Autophagy_Autophagy
21. Apoptosis_Anti-apoptosis mediated by external signals via NF-kB
22. Cell adhesion_Attractive and repulsive receptors
23. Apoptosis_Apoptosis stimulation by external signals
24. Cell adhesion_Synaptic contact
25. Development_EMT_Regulation of epithelial-to-mesenchymal transition
26. Signal transduction_NOTCH signaling
27. Apoptosis_Anti-Apoptosis mediated by external signals by Estrogen
28. Protein folding_Response to unfolded proteins
29. Reproduction_GnRH signaling pathway
30. Cardiac development_Role of NADPH oxidase and ROS
31. Development_Hemopoiesis, Erythropoietin pathway
32. Inflammation_IgE signaling
33. Immune response_TCR signaling
34. Cell adhesion_Platelet-endothelium-leucocyte interactions
35. Immune response_Phagosome in antigen presentation
36. Apoptosis_Apoptotic mitochondria
37. Apoptosis_Anti-Apoptosis mediated by external signals via MAPK and JAK/STAT
38. Signal transduction_ESR1-nuclear pathway
39. Proliferation_Negative regulation of cell proliferation
40. Inflammation_Neutrophil activation
41. Cardiac development_Wnt_beta-catenin, Notch, VEGF, IP3 and integrin signaling
42. Signal Transduction_TGF-beta, GDF and Activin signaling
43. Cardiac development_BMP_TGF_beta_signaling
44. Transport_Calcium transport
45. Development_Ossification and bone remodeling
46. Inflammation_IL-10 anti-inflammatory response
47. Signal transduction_Nitric oxide signaling
48. Reproduction_Gonadotropin regulation
49. Protein folding_Protein folding nucleus
50. Signal transduction_CREM pathway

Figure S3

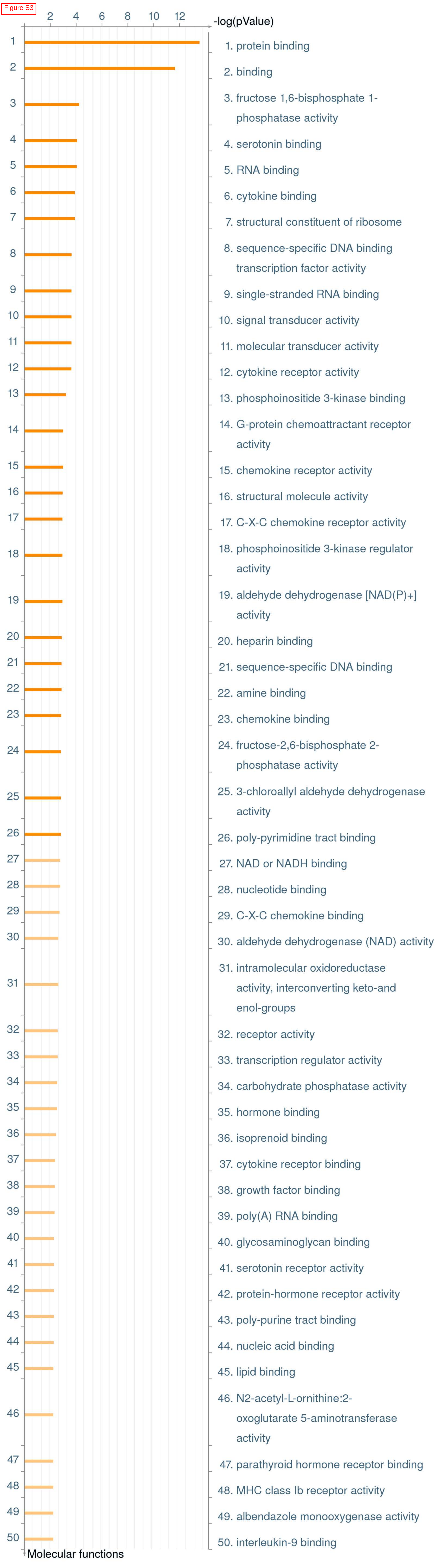
2 4 6 8 10 12
-log(pValue)
1. protein binding
2. binding
3. fructose 1,6-bisphosphate 1-phosphatase activity
4. serotonin binding
5. RNA binding
6. cytokine binding
7. structural constituent of ribosome
8. sequence-specific DNA binding transcription factor activity
9. single-stranded RNA binding
10. signal transducer activity
11. molecular transducer activity
12. cytokine receptor activity
13. phosphoinositide 3-kinase binding
14. G-protein chemoattractant receptor activity
15. chemokine receptor activity
16. structural molecule activity
17. C-X-C chemokine receptor activity
18. phosphoinositide 3-kinase regulator activity
19. aldehyde dehydrogenase [NAD(P)+] activity
20. heparin binding
21. sequence-specific DNA binding
22. amine binding
23. chemokine binding
24. fructose-2,6-bisphosphate 2-phosphatase activity
25. 3-chloroallyl aldehyde dehydrogenase activity
26. poly-pyrimidine tract binding
27. NAD or NADH binding
28. nucleotide binding
29. C-X-C chemokine binding
30. aldehyde dehydrogenase (NAD) activity
31. intramolecular oxidoreductase activity, interconverting keto-and enol-groups
32. receptor activity
33. transcription regulator activity
34. carbohydrate phosphatase activity
35. hormone binding
36. isoprenoid binding
37. cytokine receptor binding
38. growth factor binding
39. poly(A) RNA binding
40. glycosaminoglycan binding
41. serotonin receptor activity
42. protein-hormone receptor activity
43. poly-purine tract binding
44. nucleic acid binding
45. lipid binding
46. N2-acetyl-L-ornithine:2-oxoglutarate 5-aminotransferase activity
47. parathyroid hormone receptor binding
48. MHC class Ib receptor activity
49. albendazole monooxygenase activity
50. interleukin-9 binding
Molecular functions

Figure S4

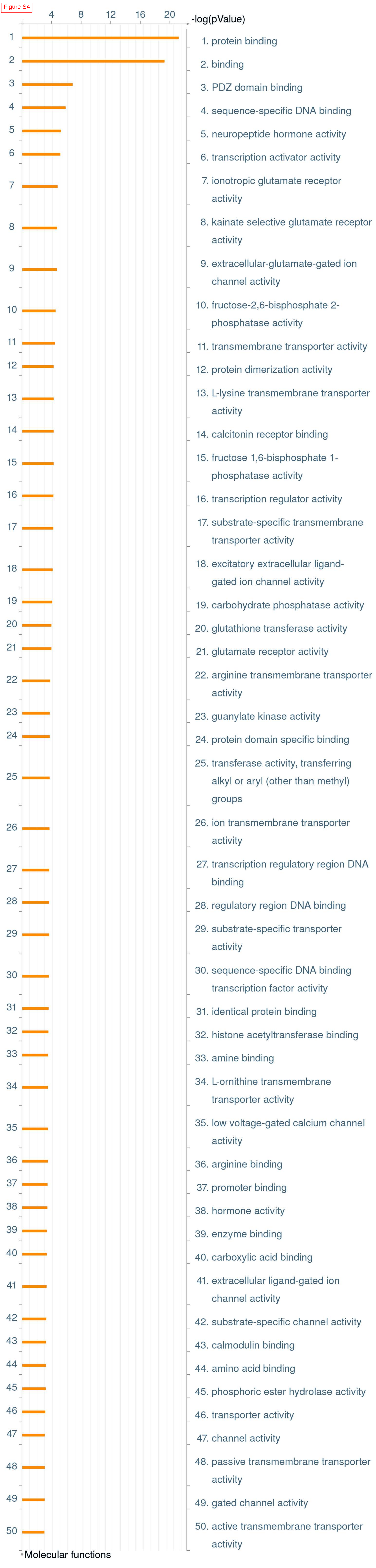
4
8
12
16
20
-log(pValue)
1. protein binding
2. binding
3. PDZ domain binding
4. sequence-specific DNA binding
5. neuropeptide hormone activity
6. transcription activator activity
7. ionotropic glutamate receptor activity
8. kainate selective glutamate receptor activity
9. extracellular-glutamate-gated ion channel activity
10. fructose-2,6-bisphosphate 2-phosphatase activity
11. transmembrane transporter activity
12. protein dimerization activity
13. L-lysine transmembrane transporter activity
14. calcitonin receptor binding
15. fructose 1,6-bisphosphate 1-phosphatase activity
16. transcription regulator activity
17. substrate-specific transmembrane transporter activity
18. excitatory extracellular ligand-gated ion channel activity
19. carbohydrate phosphatase activity
20. glutathione transferase activity
21. glutamate receptor activity
22. arginine transmembrane transporter activity
23. guanylate kinase activity
24. protein domain specific binding
25. transferase activity, transferring alkyl or aryl (other than methyl) groups
26. ion transmembrane transporter activity
27. transcription regulatory region DNA binding
28. regulatory region DNA binding
29. substrate-specific transporter activity
30. sequence-specific DNA binding transcription factor activity
31. identical protein binding
32. histone acetyltransferase binding
33. amine binding
34. L-ornithine transmembrane transporter activity
35. low voltage-gated calcium channel activity
36. arginine binding
37. promoter binding
38. hormone activity
39. enzyme binding
40. carboxylic acid binding
41. extracellular ligand-gated ion channel activity
42. substrate-specific channel activity
43. calmodulin binding
44. amino acid binding
45. phosphoric ester hydrolase activity
46. transporter activity
47. channel activity
48. passive transmembrane transporter activity
49. gated channel activity
50. active transmembrane transporter activity
Molecular functions

Figure S5

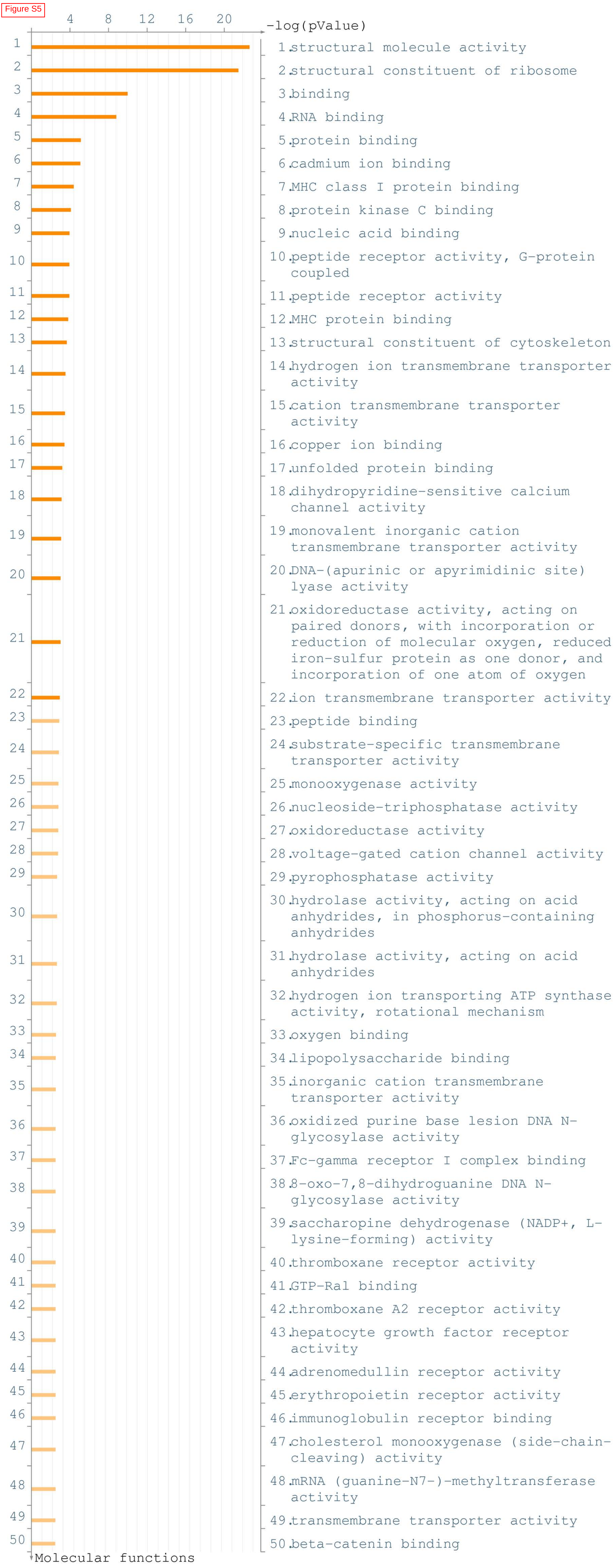
-log(pValue)
4
8
12
16
20
1.structural molecule activity
2.structural constituent of ribosome
3.binding
4.RNA binding
5.protein binding
6.cadmium ion binding
7.MHC class I protein binding
8.protein kinase C binding
9.nucleic acid binding
10.peptide receptor activity, G-protein coupled
11.peptide receptor activity
12.MHC protein binding
13.structural constituent of cytoskeleton
14.hydrogen ion transmembrane transporter activity
15.cation transmembrane transporter activity
16.copper ion binding
17.unfolded protein binding
18.dihydropyridine-sensitive calcium channel activity
19.monovalent inorganic cation transmembrane transporter activity
20.DNA-(apurinic or apyrimidinic site) lyase activity
21.oxidoreductase activity, acting on paired donors, with incorporation or reduction of molecular oxygen, reduced iron-sulfur protein as one donor, and incorporation of one atom of oxygen
22.ion transmembrane transporter activity
23.peptide binding
24.substrate-specific transmembrane transporter activity
25.monooxygenase activity
26.nucleoside-triphosphatase activity
27.oxidoreductase activity
28.voltage-gated cation channel activity
29.pyrophosphatase activity
30.hydrolase activity, acting on acid anhydrides, in phosphorus-containing anhydrides
31.hydrolase activity, acting on acid anhydrides
32.hydrogen ion transporting ATP synthase activity, rotational mechanism
33.oxygen binding
34.lipopolysaccharide binding
35.inorganic cation transmembrane transporter activity
36.oxidized purine base lesion DNA N-glycosylase activity
37.Fc-gamma receptor I complex binding
38.8-oxo-7,8-dihydroguanine DNA N-glycosylase activity
39.saccharopine dehydrogenase (NADP+, L-lysine-forming) activity
40.thromboxane receptor activity
41.GTP-Ral binding
42.thromboxane A2 receptor activity
43.hepatocyte growth factor receptor activity
44.adrenomedullin receptor activity
45.erythropoietin receptor activity
46.immunoglobulin receptor binding
47.cholesterol monooxygenase (side-chain-cleaving) activity
48.mRNA (guanine-N7-)-methyltransferase activity
49.transmembrane transporter activity
50.beta-catenin binding
Molecular functions

Figure S6

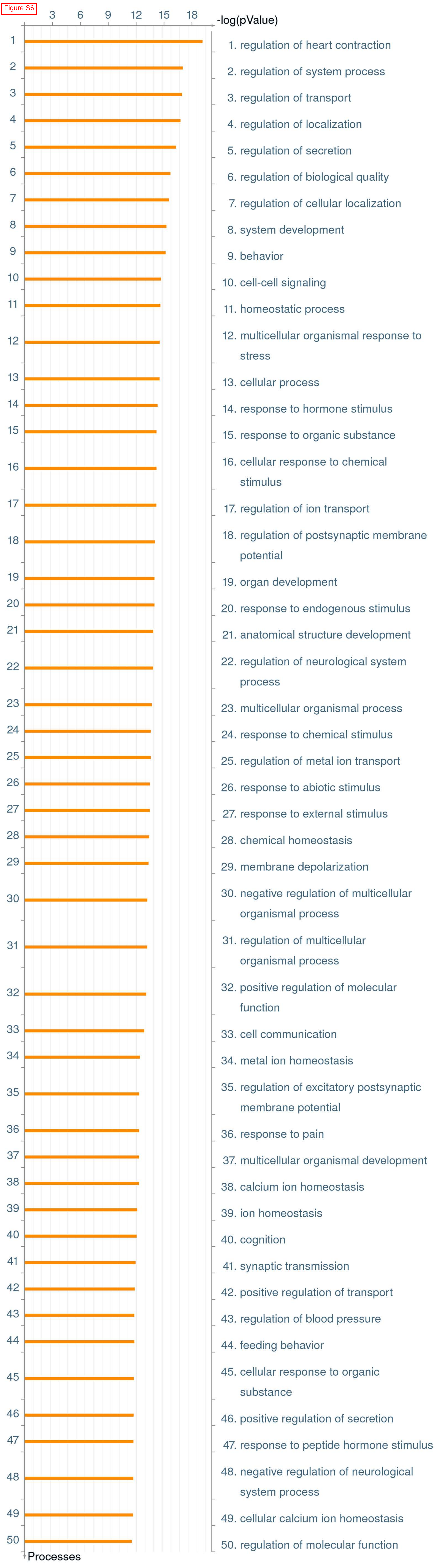

3
6
9
12
15
18
-log(pValue)
1. regulation of heart contraction
2. regulation of system process
3. regulation of transport
4. regulation of localization
5. regulation of secretion
6. regulation of biological quality
7. regulation of cellular localization
8. system development
9. behavior
10. cell-cell signaling
11. homeostatic process
12. multicellular organismal response to stress
13. cellular process
14. response to hormone stimulus
15. response to organic substance
16. cellular response to chemical stimulus
17. regulation of ion transport
18. regulation of postsynaptic membrane potential
19. organ development
20. response to endogenous stimulus
21. anatomical structure development
22. regulation of neurological system process
23. multicellular organismal process
24. response to chemical stimulus
25. regulation of metal ion transport
26. response to abiotic stimulus
27. response to external stimulus
28. chemical homeostasis
29. membrane depolarization
30. negative regulation of multicellular organismal process
31. regulation of multicellular organismal process
32. positive regulation of molecular function
33. cell communication
34. metal ion homeostasis
35. regulation of excitatory postsynaptic membrane potential
36. response to pain
37. multicellular organismal development
38. calcium ion homeostasis
39. ion homeostasis
40. cognition
41. synaptic transmission
42. positive regulation of transport
43. regulation of blood pressure
44. feeding behavior
45. cellular response to organic substance
46. positive regulation of secretion
47. response to peptide hormone stimulus
48. negative regulation of neurological system process
49. cellular calcium ion homeostasis
50. regulation of molecular function
Processes

Figure S7

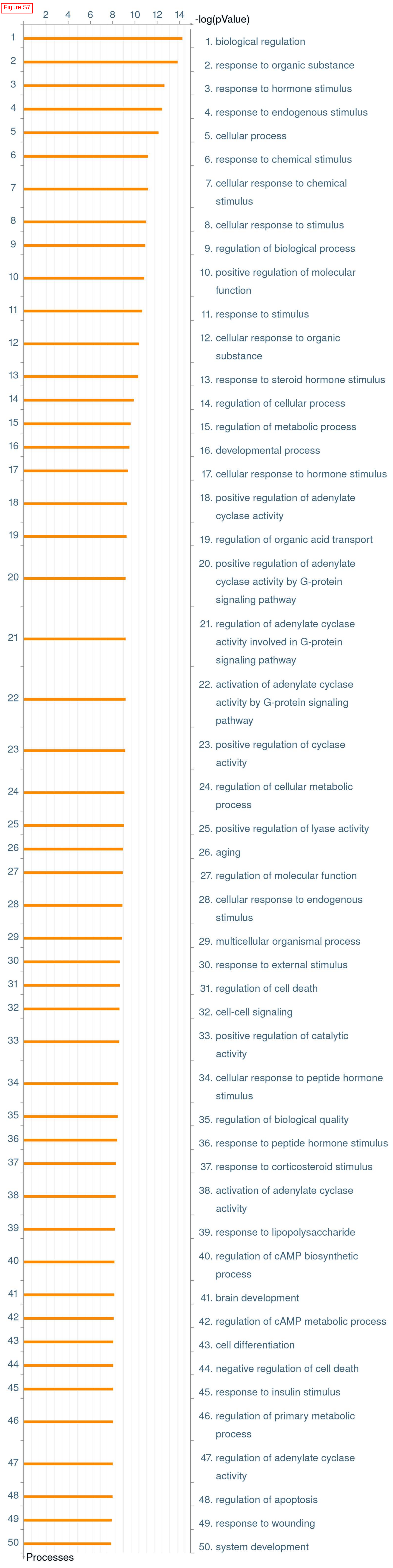
2 4 6 8 10 12 14
-log(pValue)
1. biological regulation
2. response to organic substance
3. response to hormone stimulus
4. response to endogenous stimulus
5. cellular process
6. response to chemical stimulus
7. cellular response to chemical stimulus
8. cellular response to stimulus
9. regulation of biological process
10. positive regulation of molecular function
11. response to stimulus
12. cellular response to organic substance
13. response to steroid hormone stimulus
14. regulation of cellular process
15. regulation of metabolic process
16. developmental process
17. cellular response to hormone stimulus
18. positive regulation of adenylate cyclase activity
19. regulation of organic acid transport
20. positive regulation of adenylate cyclase activity by G-protein signaling pathway
21. regulation of adenylate cyclase activity involved in G-protein signaling pathway
22. activation of adenylate cyclase activity by G-protein signaling pathway
23. positive regulation of cyclase activity
24. regulation of cellular metabolic process
25. positive regulation of lyase activity
26. aging
27. regulation of molecular function
28. cellular response to endogenous stimulus
29. multicellular organismal process
30. response to external stimulus
31. regulation of cell death
32. cell-cell signaling
33. positive regulation of catalytic activity
34. cellular response to peptide hormone stimulus
35. regulation of biological quality
36. response to peptide hormone stimulus
37. response to corticosteroid stimulus
38. activation of adenylate cyclase activity
39. response to lipopolysaccharide
40. regulation of cAMP biosynthetic process
41. brain development
42. regulation of cAMP metabolic process
43. cell differentiation
44. negative regulation of cell death
45. response to insulin stimulus
46. regulation of primary metabolic process
47. regulation of adenylate cyclase activity
48. regulation of apoptosis
49. response to wounding
50. system development
Processes

Figure S8

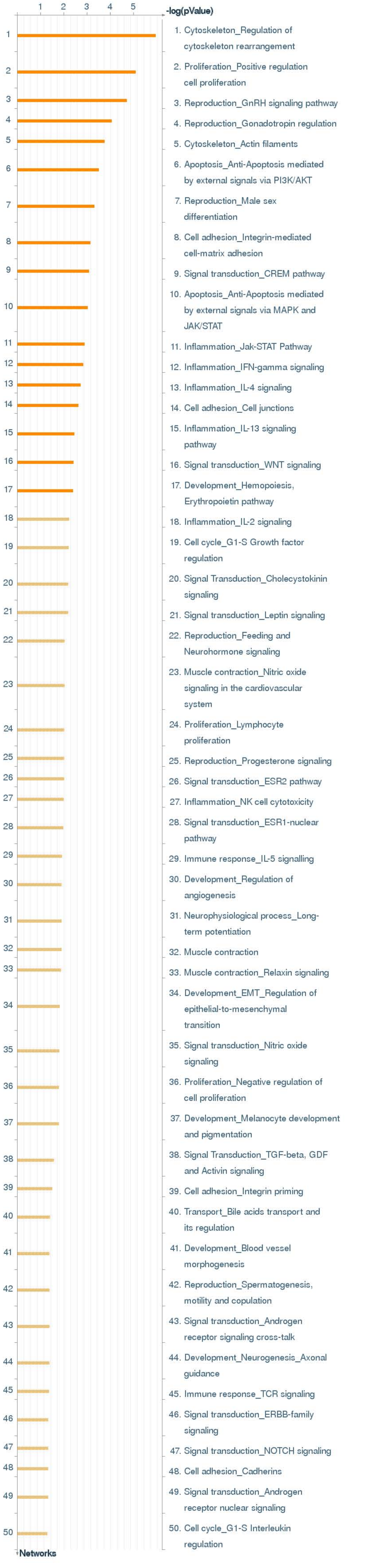

1 2 3 4 5
-log(pValue)
Networks
1. Cytoskeleton_Regulation of cytoskeleton rearrangement
2. Proliferation_Positive regulation cell proliferation
3. Reproduction_GnRH signaling pathway
4. Reproduction_Gonadotropin regulation
5. Cytoskeleton_Actin filaments
6. Apoptosis_Anti-Apoptosis mediated by external signals via PI3K/AKT
7. Reproduction_Male sex differentiation
8. Cell adhesion_Integrin-mediated cell-matrix adhesion
9. Signal transduction_CREM pathway
10. Apoptosis_Anti-Apoptosis mediated by external signals via MAPK and JAK/STAT
11. Inflammation_Jak-STAT Pathway
12. Inflammation_IFN-gamma signaling
13. Inflammation_IL-4 signaling
14. Cell adhesion_Cell junctions
15. Inflammation_IL-13 signaling pathway
16. Signal transduction_WNT signaling
17. Development_Hemopoiesis, Erythropoietin pathway
18. Inflammation_IL-2 signaling
19. Cell cycle_G1-S Growth factor regulation
20. Signal Transduction_Cholecystokinin signaling
21. Signal transduction_Leptin signaling
22. Reproduction_Feeding and Neurohormone signaling
23. Muscle contraction_Nitric oxide signaling in the cardiovascular system
24. Proliferation_Lymphocyte proliferation
25. Reproduction_Progesterone signaling
26. Signal transduction_ESR2 pathway
27. Inflammation_NK cell cytotoxicity
28. Signal transduction_ESR1-nuclear pathway
29. Immune response_IL-5 signalling
30. Development_Regulation of angiogenesis
31. Neurophysiological process_Long-term potentiation
32. Muscle contraction
33. Muscle contraction_Relaxin signaling
34. Development_EMT_Regulation of epithelial-to-mesenchymal transition
35. Signal transduction_Nitric oxide signaling
36. Proliferation_Negative regulation of cell proliferation
37. Development_Melanocyte development and pigmentation
38. Signal Transduction_TGF-beta, GDF and Activin signaling
39. Cell adhesion_Integrin priming
40. Transport_Bile acids transport and its regulation
41. Development_Blood vessel morphogenesis
42. Reproduction_Spermatogenesis, motility and copulation
43. Signal transduction_Androgen receptor signaling cross-talk
44. Development_Neurogenesis_Axonal guidance
45. Immune response_TCR signaling
46. Signal transduction_ERBB-family signaling
47. Signal transduction_NOTCH signaling
48. Cell adhesion_Cadherins
49. Signal transduction_Androgen receptor nuclear signaling
50. Cell cycle_G1-S Interleukin regulation

Figure S9

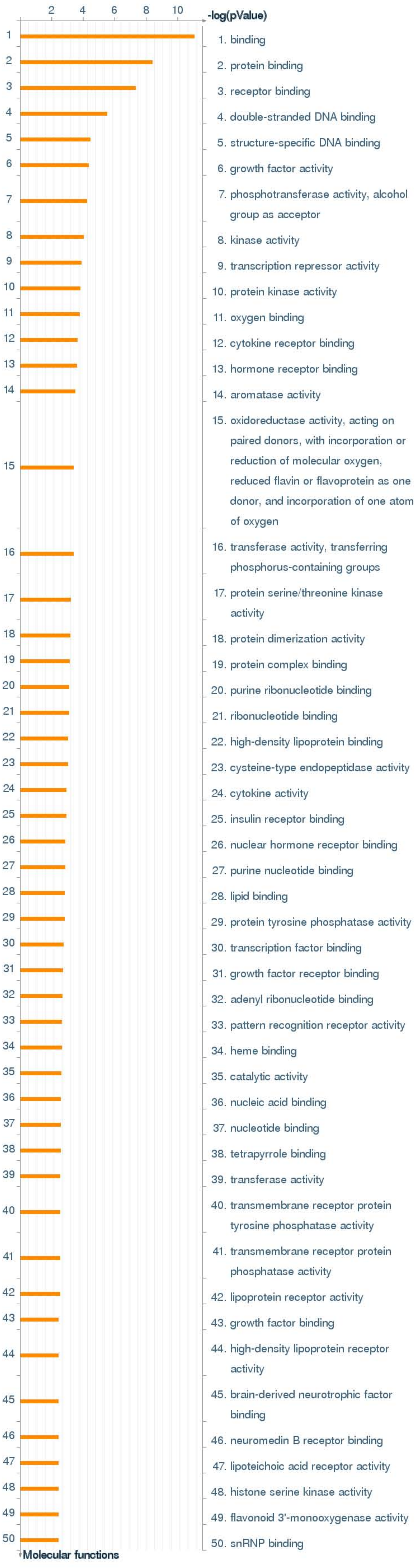

-log(pValue)
2
4
6
8
10
1. binding
2. protein binding
3. receptor binding
4. double-stranded DNA binding
5. structure-specific DNA binding
6. growth factor activity
7. phosphotransferase activity, alcohol group as acceptor
8. kinase activity
9. transcription repressor activity
10. protein kinase activity
11. oxygen binding
12. cytokine receptor binding
13. hormone receptor binding
14. aromatase activity
15. oxidoreductase activity, acting on paired donors, with incorporation or reduction of molecular oxygen, reduced flavin or flavoprotein as one donor, and incorporation of one atom of oxygen
16. transferase activity, transferring phosphorus-containing groups
17. protein serine/threonine kinase activity
18. protein dimerization activity
19. protein complex binding
20. purine ribonucleotide binding
21. ribonucleotide binding
22. high-density lipoprotein binding
23. cysteine-type endopeptidase activity
24. cytokine activity
25. insulin receptor binding
26. nuclear hormone receptor binding
27. purine nucleotide binding
28. lipid binding
29. protein tyrosine phosphatase activity
30. transcription factor binding
31. growth factor receptor binding
32. adenyl ribonucleotide binding
33. pattern recognition receptor activity
34. heme binding
35. catalytic activity
36. nucleic acid binding
37. nucleotide binding
38. tetrapyrrole binding
39. transferase activity
40. transmembrane receptor protein tyrosine phosphatase activity
41. transmembrane receptor protein phosphatase activity
42. lipoprotein receptor activity
43. growth factor binding
44. high-density lipoprotein receptor activity
45. brain-derived neurotrophic factor binding
46. neuromedin B receptor binding
47. lipoteichoic acid receptor activity
48. histone serine kinase activity
49. flavonoid 3'-monooxygenase activity
50. snRNP binding
Molecular functions

Figure S10

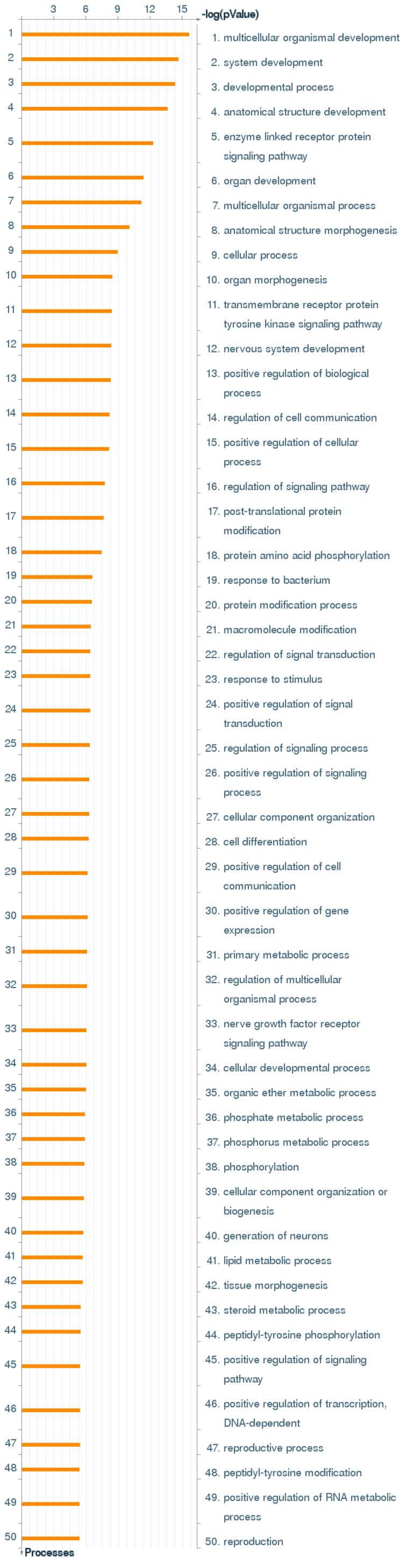

3
6
9
12
15
-log(pValue)
1. multicellular organismal development
2. system development
3. developmental process
4. anatomical structure development
5. enzyme linked receptor protein signaling pathway
6. organ development
7. multicellular organismal process
8. anatomical structure morphogenesis
9. cellular process
10. organ morphogenesis
11. transmembrane receptor protein tyrosine kinase signaling pathway
12. nervous system development
13. positive regulation of biological process
14. regulation of cell communication
15. positive regulation of cellular process
16. regulation of signaling pathway
17. post-translational protein modification
18. protein amino acid phosphorylation
19. response to bacterium
20. protein modification process
21. macromolecule modification
22. regulation of signal transduction
23. response to stimulus
24. positive regulation of signal transduction
25. regulation of signaling process
26. positive regulation of signaling process
27. cellular component organization
28. cell differentiation
29. positive regulation of cell communication
30. positive regulation of gene expression
31. primary metabolic process
32. regulation of multicellular organismal process
33. nerve growth factor receptor signaling pathway
34. cellular developmental process
35. organic ether metabolic process
36. phosphate metabolic process
37. phosphorus metabolic process
38. phosphorylation
39. cellular component organization or biogenesis
40. generation of neurons
41. lipid metabolic process
42. tissue morphogenesis
43. steroid metabolic process
44. peptidyl-tyrosine phosphorylation
45. positive regulation of signaling pathway
46. positive regulation of transcription, DNA-dependent
47. reproductive process
48. peptidyl-tyrosine modification
49. positive regulation of RNA metabolic process
50. reproduction
Processes
